# Single-cell genomics analysis reveals complex genetic interactions in an *in vivo* model of acquired BRAF inhibitor resistance

**DOI:** 10.1093/narcan/zcad061

**Published:** 2024-01-11

**Authors:** Jacob L Schillo, Charlotte R Feddersen, Rebekah M Peplinski, Lexy S Powell, Afshin Varzavand, Christopher S Stipp, Jesse D Riordan, Adam J Dupuy

**Affiliations:** Department of Anatomy & Cell Biology, Carver College of Medicine, The University of Iowa, Iowa City, IA 52242, USA; Interdisciplinary Graduate Program in Genetics, The University of Iowa, Iowa City, IA 52242, USA; Department of Anatomy & Cell Biology, Carver College of Medicine, The University of Iowa, Iowa City, IA 52242, USA; Medical Scientist Training Program, The University of Iowa, Iowa City, IA 52242, USA; Department of Anatomy & Cell Biology, Carver College of Medicine, The University of Iowa, Iowa City, IA 52242, USA; Interdisciplinary Graduate Program in Genetics, The University of Iowa, Iowa City, IA 52242, USA; Department of Anatomy & Cell Biology, Carver College of Medicine, The University of Iowa, Iowa City, IA 52242, USA; Holden Comprehensive Cancer Center, Carver College of Medicine, The University of Iowa, Iowa City, IA 52242, USA; Department of Biology, The University of Iowa, Iowa City, IA 52242, USA; Holden Comprehensive Cancer Center, Carver College of Medicine, The University of Iowa, Iowa City, IA 52242, USA; Department of Biology, The University of Iowa, Iowa City, IA 52242, USA; Department of Anatomy & Cell Biology, Carver College of Medicine, The University of Iowa, Iowa City, IA 52242, USA; Department of Anatomy & Cell Biology, Carver College of Medicine, The University of Iowa, Iowa City, IA 52242, USA; Holden Comprehensive Cancer Center, Carver College of Medicine, The University of Iowa, Iowa City, IA 52242, USA; Department of Biology, The University of Iowa, Iowa City, IA 52242, USA

## Abstract

The evolution of therapeutic resistance is a major obstacle to the success of targeted oncology drugs. While both inter- and intratumoral heterogeneity limit our ability to detect resistant subpopulations that pre-exist or emerge during treatment, our ability to analyze tumors with single-cell resolution is limited. Here, we utilized a cell-based transposon mutagenesis method to identify mechanisms of BRAF inhibitor resistance in a model of cutaneous melanoma. This screen identified overexpression of NEDD4L and VGLL3 as significant drivers of BRAF inhibitor resistance *in vivo*. In addition, we describe a novel single-cell genomics profiling method to genotype thousands of individual cells within tumors driven by transposon mutagenesis. This approach revealed a surprising genetic diversity among xenograft tumors and identified recurrent co-occurring mutations that emerge within distinct tumor subclones. Taken together, these observations reveal an unappreciated genetic complexity that drives BRAF inhibitor resistance.

## Graphical Abstract

**Graphical Abstract:**
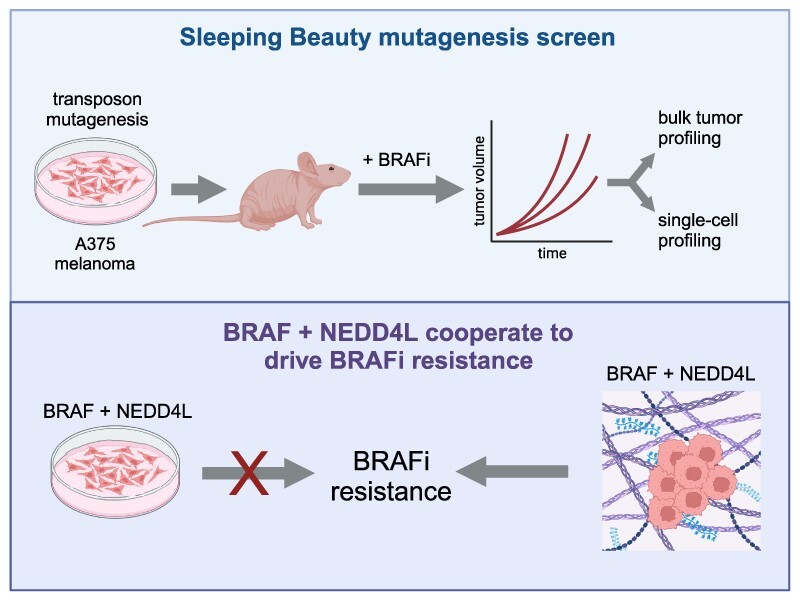

## Introduction

Improvements in genomic sequencing technology have dramatically increased our ability to describe cancer genomes, while the reduction in sequencing cost has facilitated the genomic characterization of thousands of human tumor samples. Large-scale tumor characterization has led to the identification of the most common driver events across many forms of cancer. For example, genome sequencing has revealed that ∼50% of all cutaneous melanomas harbor a mutation in the *BRAF* gene (1). Most of these events produce the BRAF^V600E^ oncoprotein, which signals in a Ras-independent fashion. This observation led to the development of several BRAF^V600E^-selective inhibitors that show clinical efficacy, particularly when paired with a MEK inhibitor (2,3). However, acquired resistance limits the long-term efficacy of BRAF/MEK inhibitors for most patients (4–8).

Previous studies have characterized mutations that are associated with acquired BRAF/MEK inhibitor (BRAFi/MEKi) resistance by sequencing matched pretreatment and progression biopsies (8,9). Several types of recurrent *BRAF* alterations have been described, including tandem duplication of the kinase domain (10), deletion of the Ras-binding domain (RBD) (9,11) and amplification of the entire *BRAF* locus (12). Although rarely observed in pretreatment biopsies, activating mutations in *Ras* genes or *NF1* loss recurrently co-occurs with the BRAF^V600E^ oncogene in BRAFi/MEKi progression samples (12,13). These mutations have been shown to maintain MAPK signaling in the presence of drug, demonstrating the reliance of melanoma cells on MAPK signaling. However, ∼10% of progression samples acquire drug resistance without reactivation of the MAPK pathway (12,14). Genetic heterogeneity also appears to play a role in acquired drug resistance as independent progression biopsies display distinct resistance mechanisms in ∼95% of patients in which multiple, independent biopsies have been evaluated (12).

The role of inter- and intratumoral genetic complexity in cutaneous melanoma treatment response has not been well characterized. In part, this is due to the challenges of obtaining sufficient tumor material to adequately capture the complexity of a patient’s disease. Ideally, both pre- and post-progression samples would be obtained from all tumor locations to fully capture the genetic complexity of an individual’s disease. The advent of circulating tumor DNA (ctDNA) analysis has provided the ability to monitor the emergence of acquired drug resistance in patients undergoing treatment (15–17), although this approach is not yet routinely applied to the clinical management of cutaneous melanoma. However, ctDNA cannot provide the cellular context in which genetic drivers of acquired drug resistance develop to reveal more complex genetic interactions that may develop within distinct tumor cell populations.

Our ability to analyze the genomes of large populations of cells in a high-throughput manner is essential for information to reach patient care, a time-dependent environment. As technologies improve to analyze tumor populations, cancer therapies and outcomes will improve. Currently, the use of single-cell technologies is cost prohibitive for many research lab settings, limiting the ability to fully characterize models of cancer. Whole-genome amplification (WGA) requires (i) isolation of individual cells, (ii) WGA of the genomic DNA and (iii) sequencing of WGA material. Given the complex nature of these steps, biological characteristics and the introduction of technical bias, there is potential to generate datasets that do not fully represent all genomic material within a cell. While possible for genomic applications, it is not feasible on the scale of current single-cell gene expression methods that can analyze thousands of cells in a single experiment. We sought to achieve a similar capability to profile Sleeping Beauty (SB) transposon insertions. Moreover, there are no available single-cell methods to provide a genome-wide profile of driver mutations in cancer cells. Here, we describe a novel forward genetics screen utilizing the SB mutagenesis system *in vivo* to monitor the genetic complexity of melanoma xenografts evolving in response to BRAF inhibition. Importantly, we describe a novel, high-throughput single-cell genomics analysis method that revealed multiple clonal populations with recurrent, distinct activating mutations simultaneously occurring within single tumors. Using this method, we discovered complex genetic interactions in drug-resistant tumors.

## Materials and methods

### Cell culture

The A375 cell line was obtained from the American Type Culture Collection (CRL-1619). Cells were cultured in Dulbecco’s modified Eagle’s medium (DMEM; Gibco, 11965-092) with 10% fetal bovine serum (FBS; Atlanta Biologicals, Premium FBS S11150) and 1× penicillin/streptomycin (Gibco, 15140-122). Cells were maintained at 37°C incubator with 5% CO_2_.

### 2D growth assay

Cells were plated in triplicate in 96-well plates at 750 cells/well in a 100 μl volume of DMEM supplemented with 10% FBS and 1× penicillin/streptomycin. The following day, resazurin assays were conducted on each well to assess the relative number of viable cells per well. After reading the assay, cells were washed with phosphate-buffered saline and 100 μl medium containing dimethyl sulfoxide (DMSO) or vemurafenib was added. Medium was changed every 3–4 days and wells were re-assayed with resazurin on day 6.

### 3D collagen growth assay

Three hundred fifty microliters of collagen I (0.8 mg/ml in DMEM; Advanced BioMatrix, 5153) was polymerized per well in 24-well tissue culture plates for 1 h at 37°C. Seven thousand five hundred cells per well were seeded in triplicate in 500 μl of DMEM supplemented with 10% FBS and 1× penicillin/streptomycin. The following day, resazurin assays were conducted to assess the relative number of viable cells per well for representative wells of each cell line. Remaining wells were treated with either DMSO or 0.5 μM vemurafenib. Medium was changed every 3–4 days and endpoint resazurin assays were conducted on days 6–9.

### Resazurin assay

A 6× solution of resazurin sodium salt (Sigma–Aldrich, R7017) resuspended in Dulbecco’s phosphate-buffered saline was added to a final concentration of 25 μg/ml in fresh DMEM and then added to multiwell plates. After a 2 h incubation at 37°C, fluorescent signal (560 nm excitation/590 nm emission) was measured using a BioTek Synergy HT plate reader. Values for wells with no cells (i.e. media only) were subtracted for each well prior to further data processing.

### Quantitative polymerase chain reaction

One microgram of total RNA was used as template for oligo-dT-primed complementary DNA (cDNA) synthesis with M-MuLV reverse transcriptase (New England Biolabs, M0253). Quantitative polymerase chain reaction (PCR) was conducted for each sample in triplicate using EvaGreen dye for detection (Biotium, 31000). Expression values for *NEDD4L* and *BRAF* were normalized to values for *TBP*.

### SB mutagenesis screen

For screen purposes, the A375 cell line was first stably transfected with the PB-EF1alpha-SB100-PGK-Puro construct and a piggyBac transposase (18) using the Qiagen Effectene Reagent (#301427). Cells were selected using 1 μg/ml puromycin (Gibco, A11138-03) over 5 days. Stably transfected cell populations were subsequently transfected with the pT2-Onc3 mutagenic transposon plasmid. Transfected cell populations were allowed to recover for ∼2 days. Athymic nude mice (Charles River, NCI ATH NU 553) were injected with 2 × 10^6^ cells in 100 μl Dulbecco’s phosphate-buffered saline (Gibco, 14190-144) subcutaneously into the left flank, right flank and subscapular locations (three tumors/mouse) with transfected cell populations. Eighty-five transposon-mutagenized xenograft tumors were engrafted and used in the genetic screen. Fifteen nonmutagenized tumors were engrafted and used in the genetic screen. Mice remained on normal chow until individual cages reached an average tumor volume of 200 mm^3^ (caliper measurement; $\frac{1}{2}$ length × width^2^). Upon an average tumor volume of 200 mm^3^ in any individual cage, that cage was randomly assigned onto BRAFi chow (Research Diets, AIN-76A Rodent Diet with 417 mg PLX4720/kg) or control chow (Research Diets, AIN-76A Rodent Diet). Tumors were harvested when any individual tumor reached a volume of 2000 mm^3^. Tumors were minced and homogenized with a razor blade and flash frozen. Tumors were placed at −80°C until prepared for genomic sequencing.

### Creation of candidate transgenes

Gene products identified from our screen were amplified from A375 cDNA: NEDD4L (NM 001144966) and VGLL3 (NM 001320493.1). Cloned cDNAs were inserted into piggyBac expression vectors that contain the human Ef1α promoter and an IRES-puromycin-polyA cassette. Stable cell lines were generated by the transfection of each applicable vector and integrated using the piggyBac transposase expression vector using the Qiagen Effectene Reagent (#301427) and subsequent selection using puromycin, 1 μg/ml over 5-day treatment. Validation of gene expression was completed by extracting RNA (Monarch Total RNA Miniprep Kit #T2010S), cDNA synthesis and quantitative reverse transcription PCR (RT-PCR) detected using a fluorescent nucleic acid dye (Biotium, EvaGreen 31000). See Supplementary Table S6 for primer sequences.

### *In vivo* validation

Athymic nude mice (Charles River, NCI ATH NU 553) were injected subcutaneously with 2 × 10^6^ A375 cells expressing a transgenic construct of empty vector, NEDD4L or VGLL3, in 100 μl Dulbecco’s phosphate-buffered saline (Gibco, 1490-144) into the left and right flanks (2 tumors/mouse, 5 mice in each cohort; for clarification, there are 10 tumors with transgenic expression of an empty vector, 10 tumors with transgenic expression of *NEDD4L* and 10 tumors with transgenic expression of *VGLL3*). The mice remained on normal chow until a cohort average tumor volume of 350 mm^3^. Upon reaching 350 mm^3^, mice were enrolled onto BRAFi chow (Research Diets, AIN-76A Rodent Diet with 417 mg PLX4720/kg) until experimental endpoint at 21 days of BRAFi chow. Tumors were harvested, minced and homogenized with a razor blade and flash frozen. Tumors were stored at −80°C for further analysis.

### Bulk sequencing

Genomic DNA was extracted from frozen samples using the GenElute™ Mammalian Genome DNA Miniprep Kit (Sigma, #G1N350-KT). DNA fragments containing transposon/genome junctions were amplified via ligation-mediated PCR as previously described (19). Samples were submitted to the the University of Iowa Institute of Human Genomics for Bioanalyzer and subsequent sequencing on the Illumina Hi-Seq 4000.

### Single-cell sequencing

Tumor nuclei were isolated following the 10x Genomics protocol (CG000167.RevA). Preparation of samples for sequencing was completed using the 10x Genomics Chromium Single Cell CNV platform protocol, in which a transposon-specific PCR is utilized in lieu of ligation with Read 2 primers. 10x Chromium Library Prep (CG000153: Single-Cell DNA Reagent Kits User Guide, Rev C) was followed as written with the following exceptions. In reference to 10x Genomics protocol CG000153.Rev3, Steps 5.1a–5.2c were omitted and replaced with primary SB PCR reaction to include 10× reaction buffer, 1 μl of 50 mM MgCl_2_, 0.5 µl of 10 mM dNTPs, 0.5 μl transposon primer, 0.5 μL Read 1 primer (see Supplementary Table S6 for sequence), Platinum Taq Polymerase and 15.9 μl H_2_O for a total volume of 25 μl. Cycle conditions were as follows:

- Step 1: 94°C for 2 min.
- Step 2: 94°C for 30 s, 55–65°C for 30 s (increase 1°C per cycle) and 72°C for 60 s; repeat 10 cycles.
- Step 3: 94°C for 30 s, 65°C for 30 s and 72°C for 60 s; repeat 25 cycles.
- Step 4: 72°C for 2 min; hold at 4°C. Resume 10x protocol at this step (CG000153.Rev3, Step 5.3a).

Following this PCR reaction, the CG000153.Rev3 protocol was resumed at Step 5.3a. Samples were submitted to the the University of Iowa Institute of Human Genomics for sequencing on the Illumina NovaSeq 6000.

### Statistics

Statistical tests are described within the figure legend of each figure.

### Permutation method to analyze co-occurrence within single-cell datasets

A set of nonredundant insertion sites was generated for each of the three single-cell datasets (Supplementary Datasets S1 and S2). We then generated 10 000 datasets in which each insertion site was replaced by a randomly selected TA dinucleotide site from the human reference genome (GRCh38). Each simulated dataset was then annotated to identify events that fell within the genes of interest (including 40 kb of promoter region). Next, the annotated random datasets were used to reconstruct the single-cell data structure to replicate the distribution of each insertion event across the cells contained within the dataset. Finally, all 10 000 simulated datasets were evaluated to determine the single co-occurrence rate of each gene pair evaluated. A raw *P*-value was determined based on this rate (e.g. 3 simulated events in 10 000 iterations = *P*-value of 0.003). A Benjamini–Hochberg correction was then used to generate an adjusted *P*-value.

## Results

### Transposon mutagenesis screen to identify drivers of BRAFi resistance *in vivo*

**Figure 1.**
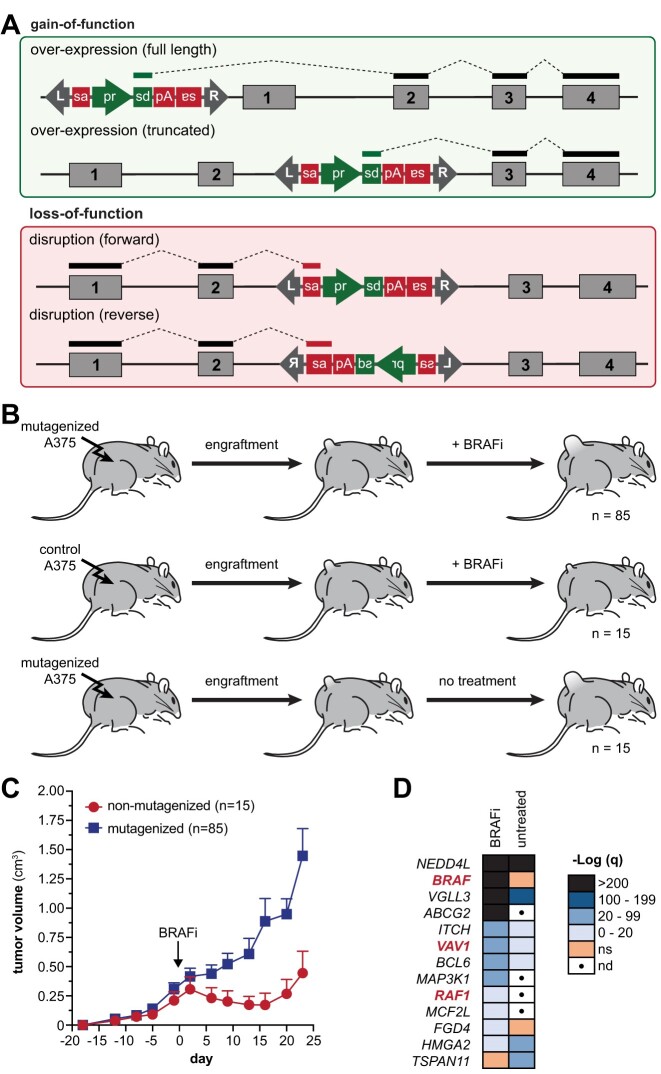
SB mutagenesis drives BRAFi resistance in a xenograft model of melanoma. (**A**) The SB transposon system can produce gain-of-function (top box) and loss-of-function (bottom box) mutations depending on the context of the transposon integration. (**B**) SB mutagenized or control A375 melanoma cells were used to generate subcutaneous xenograft tumors. Once tumors reached a volume of ∼200 mm^3^, mice were fed chow containing a BRAF inhibitor (PLX4720) or a control chow. (**C**) SB mutagenized xenograft tumors exhibited more rapid progression on treatment relative to control tumors. Measurements represent the mean tumor volume for nonmutagenized (15 tumors) and mutagenized (85 tumors) cohorts. Error bars denote standard error of the mean. The *P*-value on day 13 was calculated using a Student’s *t*-test (*P* = 0.049). (**D**) Genetic profiling of transposon-induced mutations in both BRAFi-treated and control tumors identified drivers of disease progression in either condition. Bold red font indicates genes identified in our prior cell culture screen for BRAFi resistance (24).

The SB mutagenesis system can produce both gain-of-function and loss-of-function mutations based on the location and orientation of mutagenic transposon (e.g. T2-Onc3) insertions within the host cell genome (Figure 1A) (19). Prior work has demonstrated that the SB transposase exhibits little insertion site bias when mobilizing a transposon from episomal DNA into the host genome (20). This property makes SB a useful tool for performing forward genetic screens. Genes recurrently mutated by SB transposon insertion among cells that share a common phenotype (e.g. transformed versus nonmalignant, drug-resistant versus drug-sensitive) can be tied to the phenotype since the SB transposase does not exhibit insertion site bias (20–23).

We previously described a cell-based SB screen that we used to identify BRAFi resistance drivers in cutaneous melanoma (19,24). Briefly, cultured cells are engineered to constitutively express the hyperactive SB100X transposase. A mutagenic transposon, such as T2-Onc3, is then introduced into the SB100X-expressing cells to initiate transposon mutagenesis. Cells with a desired phenotype are then isolated out of a complex population of mutagenized cells, and the transposon-induced mutations are identified within these cells. Gene mutations that are statistically enriched within the selected cell population provide a list of candidate genes that are associated with the phenotype. Evaluation of recurrent transposon insertions within each candidate gene then reveals the mechanism (i.e. overexpression, expression of a truncated gene product or disruption) through which the gene produces the desired phenotype.

Our prior work identified drivers of BRAFi resistance using cultured human melanoma cell lines (24). Here, we sought to identify drivers of BRAFi resistance *in vivo* using a similar approach. For these experiments, we utilized the *BRAF^V600E^*-mutant A375 melanoma cell line, which is sensitive to BRAF inhibitors. We created a stable population of A375 melanoma cells expressing the SB100X hyperactive transposase (A375-SB100X). The A375-SB100X cells were subsequently transfected with either an EGFP (i.e. control) or mutagenic pT2-Onc3 plasmid. Populations of control or T2-Onc3 mutagenized cells were pooled and used to generate subcutaneous xenograft tumors in athymic nude mice (Supplementary Figure S1). Once tumors acquired an average volume of 200 mm^3^, mice were transferred to cages containing either PLX4720-containing (BRAFi) chow or a control chow. Three cohorts were generated for this experiment: BRAFi-treated T2-Onc3 mutagenized xenografts, untreated T2-Onc3 mutagenized xenografts and BRAFi-treated control xenografts (i.e. nonmutagenized). Tumor volume was measured regularly until tumors surpassed a volume of 2000 mm^3^, at which time mice were euthanized and tumor material was collected.

As expected, BRAFi treatment initially caused regression of control tumors for 2–3 weeks until tumors exhibited spontaneous progression (Figure 1C). In contrast, xenograft tumors generated with T2-Onc3 mutagenized cells showed more rapid progression on BRAFi treatment (Figure 1C), indicating that SB mutagenesis is capable of driving resistance to BRAF inhibitors *in vivo*. Untreated xenograft tumors derived from mutagenized cells did not exhibit any unusual characteristics in terms of engraftment or growth rate, relative to historical A375 xenograft experiments we have previously performed (data not shown).

**Figure 2.**
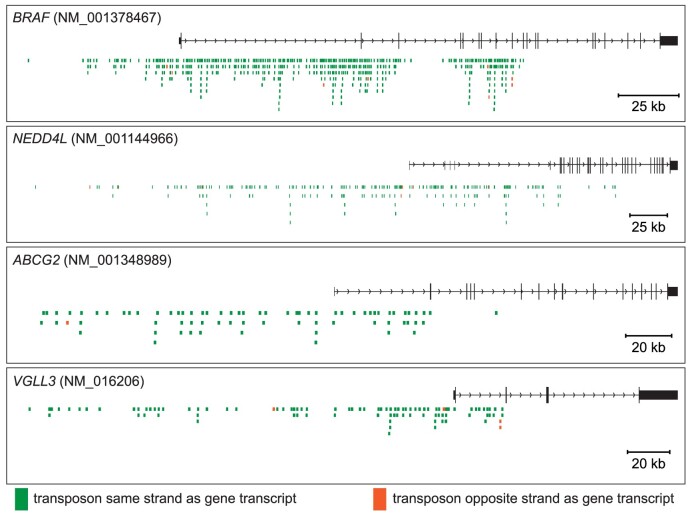
Transposon-induced overexpression of *BRAF*, *NEDD4L*, *ABCG2* and *VGLL3* is predicted to drive BRAFi resistance. Analysis of transposon insertion sites identified nonrandom clusters of transposon-induced mutations in the promoter and 5′ region of *BRAF*, *NEDD4L*, *ABCG2* and *VGLL3*. The majority of insertion events are in the same transcriptional orientation as each gene, indicative of transposon-driven overexpression (Figure 1A).

Once animals reached the endpoint, tumors were harvested and finely minced to generate a tumor slurry. This was done to obtain a more representative sample across all regions of each tumor. A DNA extraction was then performed on each individual transposon-mutagenized tumor from the BRAFi-treated and control cohorts (Figure 1B). Genomics analysis of SB mutagenized tumors revealed distinct drivers of tumor growth and/or BRAFi resistance (Figure 1D). A total of 15 distinct drivers were identified in the BRAFi-treated tumors (Supplementary Table S1). Of these, only 3 were identified in our previous culture-based screen (*BRAF*, *VAV1*, *RAF1*) (24), while 12 were unique to the xenograft model. *BRAF*, *NEDD4L*, *ABCG2* and *VGLL3* were the most frequently mutated by transposon insertions in tumors that emerged in the presence of BRAF inhibitors. The position and orientation of the insertions indicate that transposon-mediated overexpression of these genes contributes to BRAFi resistance (Figure 2). Prior studies have reported ABCG2, an ABC transporter implicated in multidrug resistance, as having potential to increase BRAFi resistance in melanoma (25–27). However, neither NEDD4L nor VGLL3 has been previously implicated in drug resistance. Transposon insertions within the *CDC27P11* (cell division cycle 27 pseudogene 11) locus were also statistically enriched in the BRAFi-treated tumors (Supplementary Table S1). Upon further inspection, we determined that the *CDC27P11* is likely an artifact due to copy number amplifications in the A375 cell line around the region of chromosome 21 where the *CDC27P11* locus resides. We did not identify additional regions of genome amplification contributing to an overestimation of significance (Supplementary Figure S2). The gCIS analysis pipeline used to perform the statistical analysis of transposon insertion site data assumes a diploid genome and therefore does not currently account for regions of copy number variants (CNVs) in tumor cell lines (19). Consequently, we did not further evaluate *CDC27P11* in this study.

Interestingly, despite observing no obvious impact of SB mutagenesis on the engraftment or growth of untreated A375 xenografts (Supplementary Figure S3), 11 recurrent driver mutations achieved statistical significance in these tumors (Figure 1D and Supplementary Table S2). This suggests that A375 cells still undergo selective pressure to generate a subcutaneous tumor in immunocompromised mice such that recurrent mutations are expanded within the xenograft tumors. Importantly, 3 of the 11 drivers (NEDD4L, VGLL3, ITCH) were also detected in the BRAFi-treated tumors. A prior study found that expression of *NEDD4L* is upregulated in melanomas relative to benign nevi, suggesting a role for NEDD4L in early-stage melanoma progression (28). Another study demonstrated that both ITCH and NEDD4L proteins interact with BRAF (29). While ITCH mediates polyubiquitination of BRAF, the functional consequence of the NEDD4L–BRAF protein interaction is unclear (29). Based on these observations, we chose to evaluate the role of *NEDD4L* in mediating BRAFi resistance.

### Validation of candidate BRAFi resistance drivers

The strongest candidate drug resistance drivers from our *in vivo* transposon mutagenesis screen were *BRAF* and *NEDD4L* (Figure 1D). We observed 386 distinct insertion sites across 77 of 85 tumors within the *BRAF* locus (Supplementary Table S1). The distribution of the insertion sites within the *BRAF* locus revealed two distinct peaks: a cluster in the 5′ region of the gene and a second cluster upstream of exon 9 (Figure 2). It is important to note that the A375 melanoma cell line is homozygous for BRAF^V600E^, so all transposon insertion events impact expression of the oncogenic form of BRAF.

The significance of the distinct cluster regions within *BRAF* can be explained by the mechanism of action of monomeric BRAF^V600E^ inhibitors. Previous work has shown that while monomeric BRAF^V600E^ can be bound by selective BRAF inhibitors (11), dimerization of BRAF^V600E^ prevents one BRAF^V600E^ protomer from binding drug (11). Thus, Ras-dependent dimerization of BRAF^V600E^ can increase resistance to BRAFi treatment by increasing dimerization of full-length BRAF^V600E^. The N-terminal truncation of BRAF^V600E^ deletes the RBD, allowing Ras-independent dimerization of BRAF^V600E^ and increased drug resistance regardless of upstream Ras signaling (11).

This is an important distinction in understanding the mechanism of drug resistance mediated by genomic alterations of the *BRAF* locus. Amplification of the *BRAF* locus is associated with drug resistance in ∼17% of patients (12). Drug resistance in this context is presumably dependent on upstream Ras signaling to drive homodimerization of BRAF^V600E^. Conversely, deletion of the BRAF^V600E^ RBD, observed in ∼28% of patient cases (12), is not reliant on Ras signaling to drive drug resistance.

Our prior work has shown that the cluster upstream of exon 9 drives expression of a truncated form of BRAF^V600E^ lacking the RBD (24). The cluster in the 5′ region of *BRAF* includes the promoter region as well as the first two large introns of the gene (Figure 2). This cluster is predicted to drive overexpression of full-length BRAF^V600E^, mimicking a gene amplification event. Importantly, the cluster at the *BRAF* 5′ region is not commonly observed in our cell culture screen and is instead a unique feature of the *in vivo* screen (24).

The next strongest candidates from our *in vivo* drug resistance screen were *NEDD4L* and *VGLL3*. Transposon integrations within the *NEDD4L* locus are spread throughout the promoter and first introns of *NEDD4L* in the same transcriptional orientation to overexpress full-length or near-full-length NEDD4L transcripts (Figure 2). This indicates that simple overexpression of NEDD4L is associated with increased BRAFi resistance. Like *NEDD4L*, transposon insertions around the *VGLL3* locus are clustered in the 5′ promoter region and first intron of the gene, again indicating that transposon-driven overexpression of VGLL3 is associated with increased drug resistance (Figure 2).

**Figure 3.**
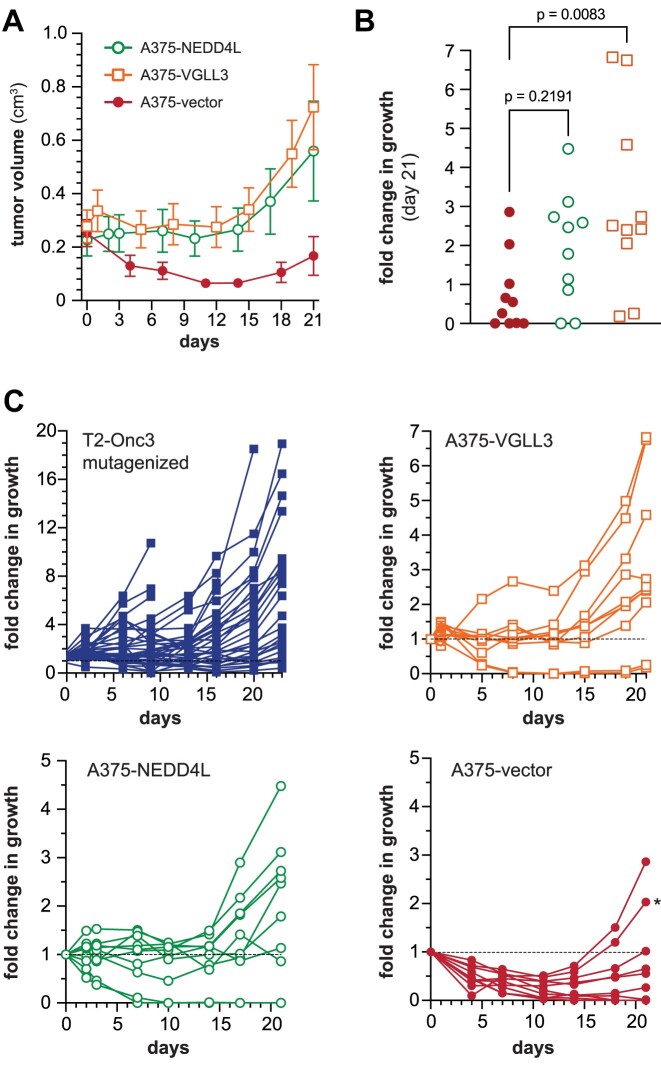
NEDD4L and VGLL3 drive resistance to BRAF inhibitors *in vivo*. Subcutaneous xenograft tumors were established using A375 cells overexpressing NEDD4L, VGLL3 or empty vector (**A**). Once tumors were established, mice were fed chow containing PLX4720 until tumors reached the experimental endpoint. (**B**) The relative fold change in tumor growth is shown at the endpoint on day 21. An ANOVA test shows that VGLL3-expressing tumors exhibit more growth than control tumors, while NEDD4L-expressing tumors do not. (**C**) The growth profiles of individual tumors are shown for each cohort. The growth of each tumor is shown as a fold change relative to its volume at the start of treatment (i.e. day 0). The T2-Onc3 mutagenized cohort described in Figure 1 is provided for relative comparison. The asterisk indicates a control tumor that was subsequently verified to express a truncated BRAF^V600E^ protein verified by western blotting (Supplementary Figure S4).

We generated transgenes to overexpress full-length NEDD4L and VGLL3 in A375 cells. Next, we established subcutaneous tumors expressing either candidate gene or an empty vector control. Cohorts were fed BRAFi-containing chow once tumors reached an average volume of 350 mm^3^ (Figure 3). As expected, control tumors decreased in volume in response to treatment for 2 weeks. Beyond that time point, control tumors grow in the presence of the drug, due to the acquisition of spontaneous resistance. We confirmed the spontaneous acquisition of a BRAF truncation in one of the control tumors that eventually progressed on treatment (Figure 3C and Supplementary Figure S4). The VGLL3-expressing tumors showed significantly increased growth in the presence of BRAFi treatment, providing validation of VGLL3 as a BRAFi resistance driver (Figure 3B). Overexpression of NEDD4L showed a trend of increased growth at the endpoint, although the difference in tumor growth was not statistically significant. In the case of both candidate genes, overexpression produced a period of stable disease that was followed by increased growth after ∼2 weeks of treatment (Figure 3C).

### Single-cell profiling of transposon insertion sites within drug-resistant tumors

Despite the high *NEDD4L* mutation frequency in BRAFi-treated tumors in our screen (Figure 1D), NEDD4L overexpression alone provided a modest increase in tumor growth in the presence of BRAFi treatment *in vivo* (Figure 3B). While we were able to validate VGLL3 as an *in vivo* driver of BRAFi resistance, its overexpression was not able to produce the growth of the most aggressive T2-Onc3 mutagenized tumors from the genetic screen (Figure 3C). It is possible that genetic heterogeneity and more complex interactions among co-occurring transposon-induced mutations could explain the more robust growth among the mutagenized xenograft cohort (Figure 3C). For instance, while 85 independent transposon-mutagenized xenograft tumors were analyzed from the *in vivo* BRAFi resistance screen, 386 independent insertion sites were detected in these tumors within the *BRAF* locus (>5 sites per tumor on average). The presence of multiple, independent transposon insertions within a single gene suggests the presence of independent subclones within each tumor. This raises the distinct possibility that genetic cooperativity between independent transposon-induced mutations contributes to BRAFi resistance within individual tumor subclones. More specifically, overexpression of NEDD4L and/or VGLL3 may rely on one or more additional transposon-induced mutations to drive drug resistance *in vivo*. Unfortunately, our current method of profiling transposon insertion sites relies on genomic DNA from a bulk tumor sample and therefore cannot resolve the clonal architecture of a complex tumor sample.

We sought to develop a single-cell analysis method to address this technical limitation. There are limited existing single-cell methods that provide the ability to obtain genomic information. Among the available methods, we determined that the single-cell CNV method developed by 10x Genomics offered an opportunity to generate transposon insertion profiles at the single-cell level (Supplementary Figure S5). Briefly, the single-cell CNV method generates a hexamer-primed library of genomic fragments, each labeled with a distinct cell barcode added to one side of each amplicon. We designed a transposon-specific primer tagged with the adaptor corresponding to one adaptor of the single-cell CNV workflow (i.e. Read 2). This primer was used in combination with the standard primer (i.e. Read 1). PCR amplification using these primers enables the amplification of cell-barcoded transposon junction sequences that can be individually assigned to a specific cell.

Three independent mutagenized BRAFi-treated tumor samples were processed to isolate individual nuclei following the 10x Genomics recommended protocol (CG000167.RevA) (Supplementary Figure S6). Nuclei were then combined with the single-cell reagents on a Chromium device to create thousands of individual cell bead emulsions that were then treated to denature the genomic DNA. An isothermal incubation step then generates barcoded genomic DNA fragments using random hexamer primers. These fragments are then ligated to a unique cell barcode that also contains a common adaptor sequence for subsequent amplification steps. At this stage, the emulsions were disrupted, and the barcoded genomic fragments were collected. We next performed a modified PCR strategy to specifically amplify genomic fragments containing the transposon junction sequences. The resulting amplicons were then purified and directly sequenced on an Illumina NovaSeq 6000.

We built a custom sequence analysis pipeline to specifically analyze the transposon junction sequences that were present within each of the three samples we processed for single-cell analysis. Briefly, each read was first analyzed to identify the 16-base cell barcode, and the read header was modified to contain the cell barcode. Reads containing the transposon tag were next isolated and trimmed to remove all adaptor and transposon sequences. The resulting genomic sequence was then mapped to the human reference genome. The mapping data were then used to compile information on all cells present within each sample (Supplementary Datasets S1–S3). Poor quality cells (<2 insertion sites with >9 reads per site) were removed from each dataset to generate a final set of cells with their corresponding transposon insertion sites. Next, an adjacency matrix of transposon insertion sites was generated for each sample to record the number of cells harboring each pair of unique insertion events observed within each sample (Supplementary Tables S3–S5). Subclones were then identified by performing hierarchical clustering of the insertion site adjacency matrix to identify groups of co-occurring insertion sites present within each tumor. Finally, we identified high-quality cells that could be uniquely assigned to a distinct cluster within each sample.

**Figure 4.**
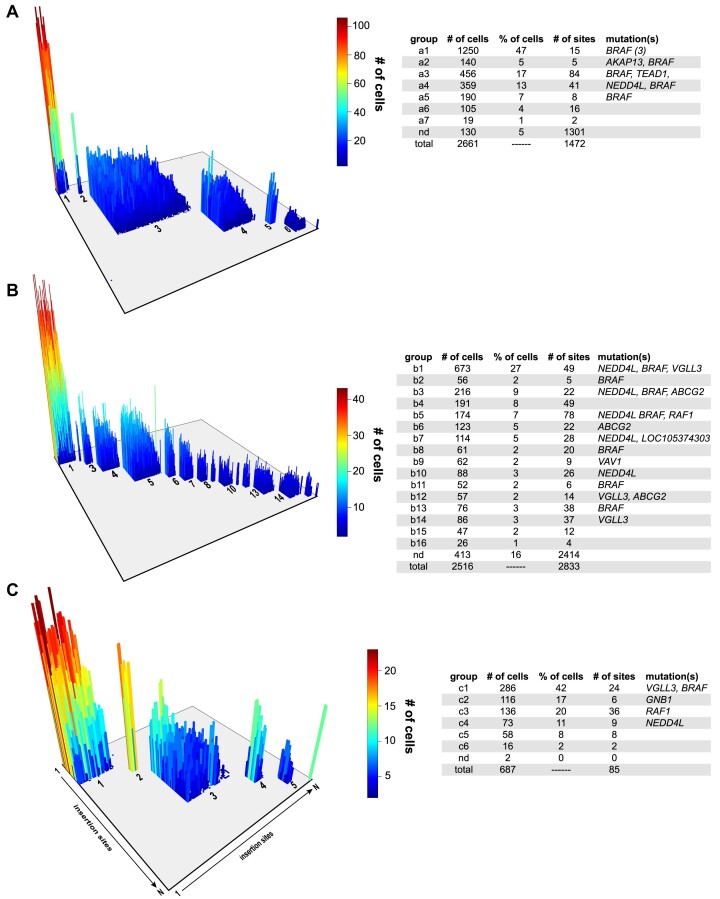
Single-cell analysis of transposon insertion sites in an A375 xenograft model of acquired BRAFi resistance. A variation of the 10x Genomics CNV workflow was used to profile transposon insertion sites in single cells isolated from three independent BRAFi-resistant melanoma xenografts (**A**–**C**). A three-dimensional rendering shows an adjacency matrix of insertion sites detected in each sample. Clusters of co-occurring transposon insertion events were identified using hierarchical clustering. The *z*-axis indicates the number of cells harboring each pair of transposon insertion events. The adjacent tables provide details on the independent subclones detected within each tumor, including a list of affected genes within each.

The results of our single-cell transposon insertion site analysis confirmed the presence of between 6 and 16 distinct subclones of cells within each of the three BRAFi-treated tumors (Figure 4). These clusters are defined by entirely nonoverlapping sets of transposon insertion events, supporting an independent origin for each cluster. The number of cells detected within each cluster was highly variable, ranging between 19 and 1250 cells. In addition, the number of unique transposon-induced mutations within each cluster was also highly variable (2–84 sites). Additionally, greater sequencing coverage is needed to further resolve subclonal architecture. In all three tumors, a small percentage of cells could not be placed within a distinct cluster (0.3–16%). Some of these events were likely caused by technical artifacts in which a single emulsion contained two or more nuclei, thus creating a ‘cell’ that mapped to multiple clusters. However, most unassigned cells (∼97% of the 556 unassigned cells) harbored entirely unique transposon insertion sites observed in only a single cell, suggesting substantial genetic complexity within each tumor.

We next evaluated the independent transposon insertion events to determine which BRAFi drivers were present within each subclone. For this analysis, we looked for transposon insertions within genes that had a false discovery rate ≤0.05 based on the analysis of bulk tumor samples (Figure 1D and Supplementary Table S1). A single BRAFi driver was identified in 13 of the 29 subclones, while 9 carried two or more driver events. Seven subclones lacked insertions in the BRAFi driver genes identified by analysis of the bulk tumors. These subclones tend to represent the smallest cell clusters within each of the samples, although there were exceptions to this trend. For example, subclone b4 contained 191 cells and is defined by 49 distinct transposon insertions. However, none of these insertion events lie within a BRAFi resistance driver identified by analysis of the bulk tumors (Figure 4B).

The main objective of the single-cell analysis was to determine whether more complex genetic interactions were occurring between different BRAFi resistance drivers. Toward this end, we identified tumors from bulk sequencing that exhibited diversity among the major candidate drivers of resistance to provide greater statistical power to identify recurrent gene interactions. We detected recurrent, co-occurring transposon insertions in both *BRAF* and *NEDD4L* loci in four independent subclones (a4, b1, b3, b5) in two tumors (Figure 4). No other instances of recurrent co-occurring driver events were observed within the identified clusters. It is important to note that while three of the four subclones harboring both *BRAF* and *NEDD4L* insertions were detected within the same tumor mass (Figure 4B), each subclone is defined by completely independent transposon insertion events. This observation strongly suggests that all four subclones acquired co-occurring insertions within *BRAF* and *NEDD4L* independently (Supplementary Figure S7).

The results of our single-cell analysis suggested that transposon-driven overexpression of NEDD4L precedes overexpression of full-length BRAF (Figure 4). Based on this trend, we re-evaluated the results of our initial *in vivo* validation experiment in which NEDD4L was overexpressed in A375 xenografts (Figure 3). As we previously noted, NEDD4L overexpression appears to provide some protection from drug during the first 2 weeks of treatment (Figure 3). The NEDD4L xenografts also exhibit more rapid progression compared to control A375 xenografts during the third week of treatment. We hypothesized that the progression of NEDD4L xenografts is driven by spontaneous overexpression of BRAF^V600E^. Quantitative RT-PCR confirmed that NEDD4L xenografts show a significant increase in BRAF^V600E^ expression relative to the A375 control tumors (Supplementary Figure S8). In contrast, BRAF^V600E^ expression in VGLL3 transgenic tumors was not significantly increased, nor did we detect multiple co-occurring transposon insertions within the *BRAF* and *VGLL3* loci (Figure 4). This suggests that BRAF overexpression, perhaps through amplification of the *BRAF* locus, is selected more often in the context of NEDD4L overexpression. This observation provides additional experimental evidence that increased expression of NEDD4L cooperates with BRAF^V600E^ overexpression to drive BRAFi resistance *in vivo*.

### Analysis of co-occurring transposon-induced mutations in BRAFi-resistant tumors

The single-cell analysis of three independent BRAFi-resistant tumors revealed several important observations. First, each tumor contained multiple, genetically distinct clusters of cells harboring nonoverlapping transposon-induced mutations (Figure 4). Hundreds of individual cells were also detected in two of the three tumors, in addition to the clusters (Figure 4A and B). Finally, the presence of recurrent, co-occurring insertions within the *BRAF* and *NEDD4L* loci provided an indication that more complex interactions between independent transposon-induced mutations could be contributing to the emergence of BRAFi resistance.

These observations led us to perform a more comprehensive analysis to detect other co-occurring genetic events within the three tumors analyzed by single-cell profiling. Each single-cell dataset was independently assessed to identify co-occurring transposon insertions within all annotated RefSeq genes. For this analysis, co-occurring insertion events were defined as independent insertion events within the same gene pair. Each unique insertion site within a given gene was allowed to contribute to only a single co-occurrence event to ensure that co-occurrence events within clusters of related cells are counted only once within each sample. This analysis identified 63 genes found in 56 recurrent co-occurring pairs in one or more samples. We next sought to determine the statistical significance of observing this number of recurrent pairs of transposon-induced mutations.

Due to the complex structure of the datasets, we decided to utilize a permutation method to determine the significance of the observed mutation co-occurrences. First, we generated a matched random dataset in which each transposon insertion event within a given single-cell dataset was replaced by a randomly selected TA dinucleotide site in the reference genome. Importantly, this process of random replacement maintained the structure and relationship among the cells of each sample. Next, the randomly selected insertions were annotated to identify sites within the 59 genes identified as recurrently co-mutated in the single-cell datasets. The annotated random dataset was then queried to determine the frequency of co-occurring insertions within the observed pairs of genes. This process was repeated for a total of 10 000 random trials for each dataset. Finally, a Benjamini–Hochberg adjusted *P*-value was calculated for each gene pair based on the number of co-occurring events observed in the 10 000 simulated trials (Table 1).

**Table 1.** Analysis of co-occurring transposon-induced mutations

Gene 1	Gene 2	No. of observed co-occurrences			Adj. *P*-value for single co-occurrence^a^			Est. *P*-value		
		a	b	c	a	b	c	a	b	c
*MACROD2*	***BRAF***	1	1	2	3.02E−03	3.34E−03	6.59E−04	3.02E−03	3.34E−03	4.34E−07
*RBFOX1*	***BRAF***		1	2		2.29E−03	8.30E−04		2.29E−03	6.88E−07
*PTPRD*	*WWC3*		1	2		2.58E−03	2.38E−04		2.58E−03	5.68E−08
***NEDD4L***	***BRAF***	1	3		9.56E−04	7.81E−04		9.56E−04	4.77E−10	
***NEDD4L***	***ABCG2***	1	2		9.33E−04	2.67E−04		9.33E−04	7.11E−08	
***NEDD4L***	*TENM2*	1	2		2.96E−03	2.96E−03		2.96E−03	8.79E−06	
*TENM2*	***BRAF***	1	2		1.43E−03	2.33E−03		1.43E−03	5.44E−06	
*MITF*	*LPAR1*	3			1.26E−03			2.00E−09		
*OSBPL9*	*FAM180A*	2			1.01E−03			1.02E−06		
*SH3PXD2A*	*WDR41*	2			8.40E−04			7.06E−07		
*LOC105376514*	*MITF*	2			7.20E−04			5.18E−07		
*LOC105376514*	*ATG10*	2			6.30E−04			3.97E−07		
***TEAD1***	***BRAF***	2			6.40E−04			4.10E−07		
*CPNE8*	***BRAF***	2			5.04E−04			2.54E−07		
*CPNE8*	*FBXO10*	2			4.58E−04			2.10E−07		
*ABCC4*	*ANXA4*	2			2.33E−04			5.44E−08		
*DOK6*	*NR2F1-AS1*	2			3.88E−04			1.50E−07		
*ROCK2*	*COLQ*	2			3.60E−04			1.30E−07		
*RASGRP3*	*MITF*	2			2.29E−04			5.25E−08		
*LOC101927967*	*MITF*	2			3.15E−04			9.92E−08		
*LOC101927967*	*SLC24A2*	2			2.96E−04			8.79E−08		
*LOC101927967*	*CNTNAP3B*	2			2.80E−04			7.84E−08		
*LOC101927967*	*LPAR1*	2			2.65E−04			7.04E−08		
*PLCL1*	*LOC105372948*	2			2.52E−04			6.35E−08		
*SYN3*	*HTR1F*	2			4.94E−04			2.44E−07		
*MITF*	*SLC24A2*	2			3.36E−04			1.13E−07		
*PTPN13*	*ATG10*	2			7.37E−04			5.43E−07		
*ATG10*	*NR2F1-AS1*	2			4.20E−04			1.76E−07		
*TBXAS1*	***BRAF***	2			5.60E−04			3.14E−07		
*TBXAS1*	*ST18*	2			1.68E−03			2.82E−06		
*AGBL4*	***NEDD4L***		2			2.80E−04			7.84E−08	
*ANXA9*	*ABCA11P*		2			2.52E−03			6.35E−06	
*KIN*	*LOC102724861*		2			3.86E−04			1.49E−07	
*ITPR2*	*LOC102723906*		2			5.60E−04			3.14E−07	
*MGAT4C*	***BRAF***		2			4.31E−04			1.86E−07	
*HS3ST3A1*	***NEDD4L***		2			2.24E−04			5.02E−08	
*ITGA3*	*LOC105377171*		2			3.36E−04			1.13E−07	
*ITGA3*	*GRB10*		2			3.29E−04			1.09E−07	
***NEDD4L***	*LOC102724861*		2			6.83E−04			4.66E−07	
***NEDD4L***	*ZC3H12B*		2			1.22E−03			1.48E−06	
*LOC102724861*	*TENM2*		2			1.15E−03			1.31E−06	
*LOC102724861*	*PDE1C*		2			8.00E−04			6.40E−07	
*MACROD2*	*TENM2*		2			1.41E−02			1.99E−04	
*LOC105377171*	*GRB10*		2			2.43E−04			5.93E−08	
*LOC105377180*	*KDM5C*		2			4.15E−04			1.72E−07	
*KIAA1524*	*MEGF9*		2			3.73E−04			1.39E−07	
*IGSF11*	*EYS*		2			5.08E−03			2.58E−05	
*KALRN*	***BRAF***		2			1.24E−03			1.55E−06	
*ABCA11P*	*LOC105374483*		2			1.68E−03			2.82E−06	
***ABCG2***	***BRAF***		2			5.42E−04			2.94E−07	
***ABCG2***	*NSMCE2*		2			5.89E−04			3.47E−07	
*LOC102723906*	*EYS*		2			3.38E−03			1.14E−05	
*LOC105377865*	*ZC3H12B*		2			2.14E−03			4.60E−06	
*GRB10*	*ZC3H12B*		2			6.05E−04			3.67E−07	
*CNTNAP2*	*ZC3H12B*		2			7.13E−03			5.08E−05	
*SLC26A7*	*ZFPM2-AS1*		2			4.80E−04			2.30E−07	

Genes in bold were identified as driver genes in the bulk sequence analysis.

^a^ Based on 10 000 simulated datasets in which observed insertion events were replaced with randomly selected TA sites.

The results of the permutation analysis revealed that all observed co-occurring mutations appear at a frequency that is significantly higher than expected based on the permutation test. The analysis also revealed that seven co-occurring mutations involving eight genes are present in multiple samples (Table 1). Of these eight genes, only three (*BRAF*, *NEDD4L*, *ABCG2*) were identified as significant drivers of BRAFi resistance from the analysis of bulk tumor samples. The remaining five genes (*MACROD2*, *PTPRD*, *RBFOX1*, *TENM2*, *WWC3*) were identified only by single-cell analysis. These results strongly suggest that genetic interactions between independent transposon-induced mutations drive the emergence of BRAFi resistance in our model.

### Validation of co-occurring gene pairs

**Figure 5.**
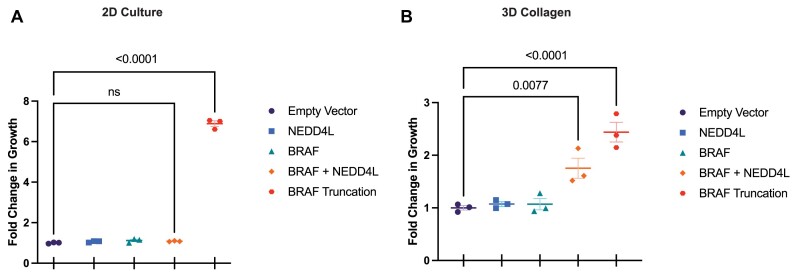
*In vitro* validation of cooperativity between NEDD4L and BRAF. A 6-day growth assay in 2D culture with A375 cells expressing transgenic candidate drivers of drug resistance to 5 μM vemurafenib (**A**). An ANOVA with multiple comparisons showed no significant increase in growth in cells co-expressing BRAF and NEDD4L. A 9-day growth assay in a 3D collagen matrix with A375 cells expressing transgenic candidate drivers of drug resistance to 0.5 μM vemurafenib (**B**). An ANOVA with multiple comparisons showed a significant increase in growth in cells co-expressing BRAF and NEDD4L (*P*= 0.0077). In 2D culture and 3D collagen assays, A375 cells expressing transgenic BRAF truncation were utilized as a positive control of growth in the presence of vemurafenib.

Based on the results of our permutation test and increased *BRAF* expression in *NEDD4L*-driven tumors, we sought to test the ability of *NEDD4L* to drive resistance when co-expressed with *BRAF*. In a 2D culture-based assay, the transgenic expression of *NEDD4L* with *BRAF* did not contribute to BRAFi resistance (Figure 5A). This is consistent with our original culture screen in which *NEDD4L* was not identified as a candidate driver of drug resistance. Conversely, when performing culture experiments on a collagen matrix, the transgenic expression of *NEDD4L* with *BRAF* significantly increased cell growth in the presence of BRAF inhibitors (Figure 5B and Supplementary Figure S9).

## Discussion

We recently described a cell-based SB transposon mutagenesis method that can be used to perform phenotype-driven screens in cultured cells (19,24). Here, we describe the first application of this method to construct a xenograft model of acquired BRAFi resistance using a human melanoma cell line. Our initial genetic analysis of bulk tumor samples identified several novel candidate genes, including NEDD4L and VGLL3 (Figure 1D). Protein interactions between NEDD4L and BRAF have been shown by two independent publications, although the functional implications of this interaction have not been well characterized (29,30). We provide additional experimental evidence that confirms the ability of NEDD4L overexpression to increase BRAFi resistance in a melanoma xenograft model (Figure 3). It has been shown that BRAF homodimerization increases resistance to monomeric BRAF^V600E^ inhibitors (11). The NEDD4L–BRAF protein interaction could mediate drug resistance through increasing the rate of BRAF homodimerization. Alternatively, NEDD4L binding to BRAF could alter the binding affinity of the BRAF inhibitor. In either case, our findings support future investigation into this mechanism, particularly considering the work of Kito *et al.*, which suggests that NEDD4L overexpression is associated with progression of benign nevi to malignant melanoma (28), and gene expression profiling by Badal *et al.*, which details upregulation of *NEDD4L* in malignant melanomas (Supplementary Figure S10) (31).

Our study is the first to implicate VGLL3 in melanoma. Genetic analysis of untreated xenograft tumors implicated VGLL3 as a novel oncogene involved in tumor growth. We also subsequently confirmed that overexpression of VGLL3 increases BRAFi resistance in A375 xenografts (Figure 3). While VGLL3 has been shown to function in the Hippo pathway, like the related family member VGLL1 (32), a role for VGLL3 in the context of cancer is not clear. However, recent genetic analyses of various forms of sarcoma show *VGLL3* gene amplifications and fusions, consistent with our identification of VGLL3 as an oncogene (33–35).

Most significantly, we describe the first genome-wide single-cell genomics method to profile transposon-induced mutations. The application of this method to our xenograft model of BRAFi resistance revealed a surprising degree of genetic complexity (Figure 4). Our results revealed two general forms of genetic complexity: the presence of multiple, independent subclones of cells harboring mutations associated with drug resistance and the novel observation of recurrent co-occurring transposon-induced mutations across different subclones and tumors.

The variation in subclone architecture was a notable feature of all three tumors subjected to single-cell analysis. The subclone number, size (i.e. number of assigned cells) and genetic complexity (i.e. number of distinct transposon insertions) varied across all three tumors (Figure 4). The variation in subclone size cannot be directly associated with relative drug resistance, meaning that the largest subclones cannot be assumed to have the greatest degree of drug resistance. This is because SB transposase is constitutively expressed within the cells, and thus transposon mutagenesis is ongoing throughout the duration of the experiment. Without longitudinal sampling of each tumor mass, we cannot approximate when each subclone emerged over the course of the drug treatment (∼3 weeks).

The genetic complexity also varied widely with some less abundant subclones exhibiting a high degree of complexity. For instance, subclones a3 and b5 were defined by 84 and 78 distinct transposon insertions, respectively (Figure 4A and B). However, these subclones showed only modest abundance within the broader bulk tumor sample. The underlying biological explanation for the variation in genetic complexity is not clear. Perhaps, these subclones are derived from epigenetically distinct cells that have different behaviors and/or drug responses. This hypothesis could be tested in the future using a single-cell method capable of capturing both genomic and transcriptomic information simultaneously. Several reports have described methods to simultaneously perform assay for transposase-accessible chromatin with sequencing and RNA sequencing on single cells (36,37). These methods could potentially be modified using a similar approach we adopted here to provide transposon insertion profiles along with gene expression data.

Perhaps, the most surprising observation from our single-cell analysis was the identification of recurrent co-occurring transposon-induced mutations (Table 1). The most frequently observed pairs of co-mutated genes were those in which one or both genes were identified as significant drivers of BRAFi resistance (Figure 1D and Supplementary Table S1). However, ∼70% of recurrent pairs of co-mutated genes involved genes that were not identified as significant through the genetic analysis of bulk tumor material. Although MITF, LPAR1 and PLCL1 have all been previously implicated in BRAFi resistance (38–40), the remaining genes shown in Table 1 have not been directly associated with drug resistance. While the role of these genes is not yet clear, it is possible that the mechanism through which they act is more context specific than the more common drivers. The appropriate context for these genes could be a specific epigenetic cell type or a combination of additional mutations with which these genes act to drive resistance.

This study focuses on an *in vivo* genetic screening approach, and as such is incapable of replicating drug resistance in all contexts. For instance, transposon insertions may better approximate the effect of gene amplification/deletion events rather than single nucleotide alterations. Nevertheless, we have demonstrated that SB mutagenesis screens can be effective at identifying pathways impacted by mutations in response to a therapeutic challenge.

Another limitation of this study is the select use of a single cell line (A375) for analysis, yet reveals significant diversity in clonal selection within a single tumor. Combinatorial alterations driving tumor evolution will likely vary across BRAF-mutant cell lines given the diversity in gene and protein expression that has been documented in melanoma cell lines. Nevertheless, our single-cell approach will facilitate the identification of complex genetic interactions in a relatively unbiased manner.

Our novel single-cell method provides a higher resolution understanding of genetic evolution of drug-resistant melanoma cells *in vivo*. However, there are many future applications of this technology one could imagine. For example, our single-cell approach could enable more detailed studies of genetic mechanisms of immunotherapy resistance using syngeneic models of cancer, and mutagenesis of nonmalignant cells could provide insight into the evolutionary adaptation during earlier stages of cellular transformation that have been difficult to study in detail due to the limited cell number in preneoplastic lesions. These are just several examples of experiments that could be possible due to the flexible nature of our cell-based transposon mutagenesis method now paired with the ability to perform single-cell genomics analysis.

## Supplementary Material

zcad061_Supplemental_Files

## Data Availability

All data are provided as supplementary figures, tables or datasets. All raw data will be provided upon request. Sequencing data have been deposited in Gene Expression Omnibus (https://www.ncbi.nlm.nih.gov/geo/) under accession number GSE232760.
