# Supplementary material for: Single-cell genomics analysis reveals complex genetic interactions in an in vivo model of acquired BRAF inhibitor resistance
Source: NAR Cancer. 2024 Jan 11;6(1):zcad061. doi: 10.1093/narcan/zcad061 (PMC10782916; doi:10.1093/narcan/zcad061)
Supplement: zcad061_Supplemental_Files [file zcad061_supplemental_files.zip › Figure_S1.pdf]

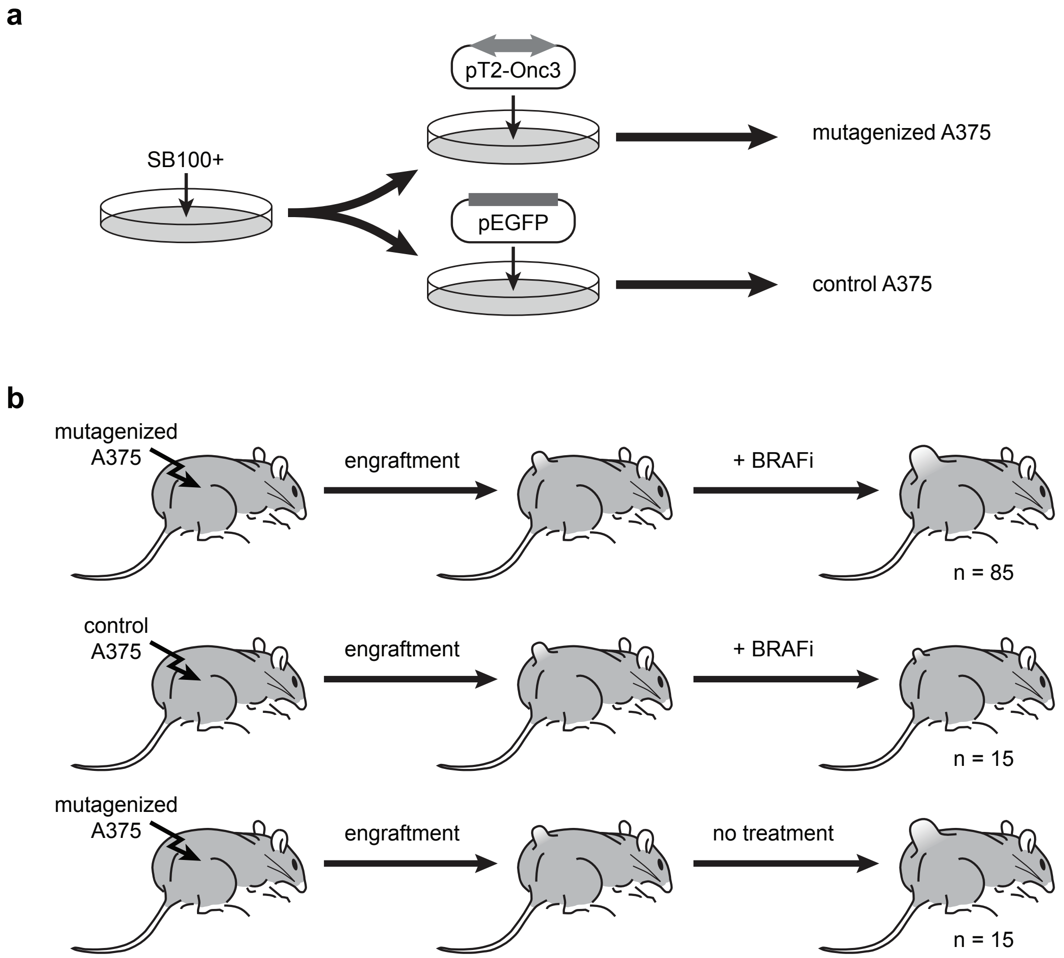

Supplemental Figure 1. Overview of Sleeping Beauty screening strategy. (**a**) A375 cells expressing the SB100X transposase were transfected with a mutagenic T2-Onc3 or a control EGFP plasmid. (**b**) Each cell population was briefly expanded in culture and then used to generate subcutaneous xenograft tumors in athymic nude mice. All mice harboring tumors generated with the control cell population were placed on chow containing the BRAF inhibitor PLX4720. Tumors generated with mutagenized cells were fed either PLX4720-containing chow or a control chow (*i.e.,* no treatment).
