# Supplementary material for: Single-cell genomics analysis reveals complex genetic interactions in an in vivo model of acquired BRAF inhibitor resistance
Source: NAR Cancer. 2024 Jan 11;6(1):zcad061. doi: 10.1093/narcan/zcad061 (PMC10782916; doi:10.1093/narcan/zcad061)
Supplement: zcad061_Supplemental_Files [file zcad061_supplemental_files.zip › Figure_S10.pdf]

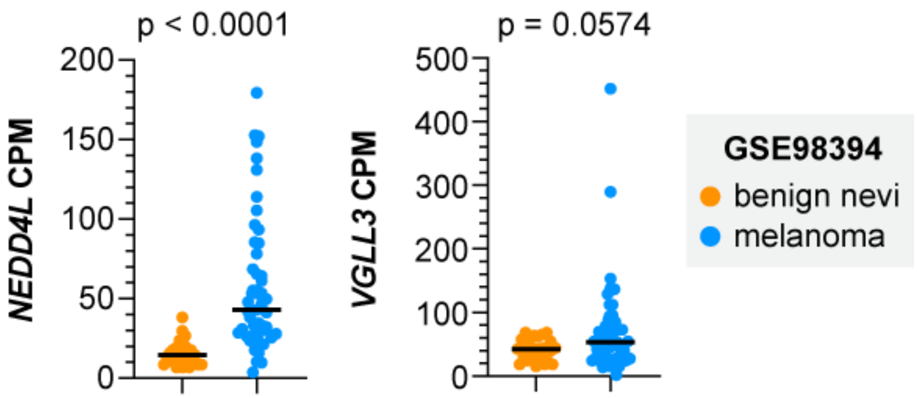

Supplementary Figure 10. Gene expression profiling performed by Badal et al. compared benign melanocytic nevi with malignant melanoma (1). This data set was evaluated for expression of *NEDD4L* (left) and *VGLL3* (right). NEDD4L expression is consistently elevated in melanoma, relative to benign nevi ($p < 0.0001$). VGLL3 expression was not significantly elevated in melanoma, relative to benign nevi ($p = 0.0574$), however several melanoma specimens exhibit substantial over-expression.

1. Badal B, Solovyov A, Di Cecilia S, . . . Celebi JT. Transcriptional dissection of melanoma identifies a high-risk subtype underlying TP53 family genes and epigenome deregulation. JCI Insight. 2017;2(9). Epub 20170504. doi: 10.1172/jci.insight.92102. PubMed PMID: 28469092; PMCID: PMC5414564.
