# Supplementary material for: Single-cell genomics analysis reveals complex genetic interactions in an in vivo model of acquired BRAF inhibitor resistance
Source: NAR Cancer. 2024 Jan 11;6(1):zcad061. doi: 10.1093/narcan/zcad061 (PMC10782916; doi:10.1093/narcan/zcad061)
Supplement: zcad061_Supplemental_Files [file zcad061_supplemental_files.zip › Figure_S2.pdf]

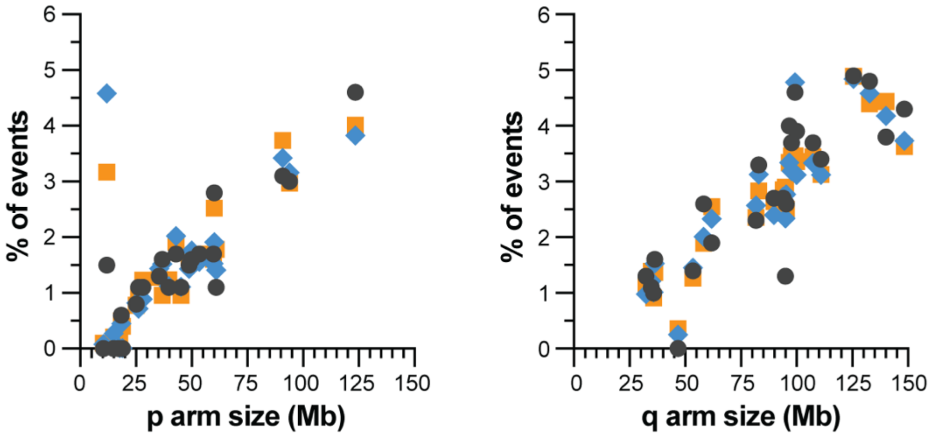

group	R^2	P value
DMSO (cell culture) ●	0.8351	<0.0001
xenografts (untreated) ■	0.6549	<0.0001
xenografts (treated) ◆	0.4308	0.0005

group	R^2	P value
DMSO (cell culture) ●	0.7092	<0.0001
xenografts (untreated) ■	0.8457	<0.0001
xenografts (treated) ◆	0.7791	<0.0001

Supplemental Figure 2. Genome-wide distribution of Sleeping Beauty transposon insertions. The normalized transposon insertion site frequencies (% of insertion events) are shown for the p arms (left) and q arms(right) for each chromosome. A Pearson correlation showed significant correlations between insertion site frequencies and chromosome arm size. The DMSO dataset is taken from a previous publication to represent unselected transposon insertion sites (23). As noted in the text, a region on the p-arm of chr21 near the *CDC27P11* locus shows an increase frequency of insertion events that we attribute to an amplification event in the A375 cell line.
