# Supplementary material for: Single-cell genomics analysis reveals complex genetic interactions in an in vivo model of acquired BRAF inhibitor resistance
Source: NAR Cancer. 2024 Jan 11;6(1):zcad061. doi: 10.1093/narcan/zcad061 (PMC10782916; doi:10.1093/narcan/zcad061)
Supplement: zcad061_Supplemental_Files [file zcad061_supplemental_files.zip › Figure_S3.pdf]

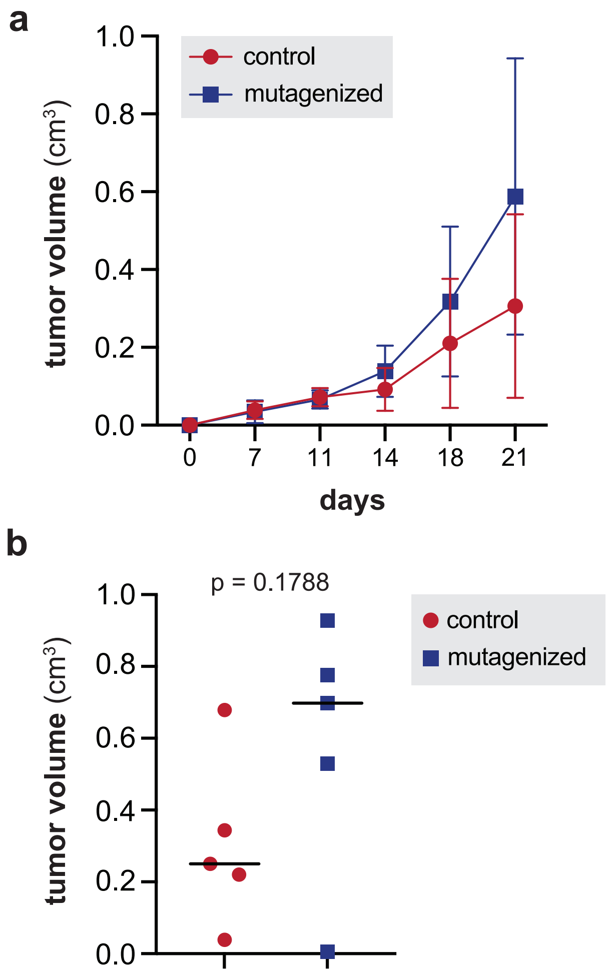

Supplemental Figure 3. Growth of tumors prior to BRAFi treatment. (**a**) SB mutagenesis did not significantly alter the growth rate of xenograft tumors relative to the control group. (**b**) The tumor volumes in the untreated mutagenized cohort were not significantly larger than the control cohort (Student's t-test).
