# Supplementary material for: Single-cell genomics analysis reveals complex genetic interactions in an in vivo model of acquired BRAF inhibitor resistance
Source: NAR Cancer. 2024 Jan 11;6(1):zcad061. doi: 10.1093/narcan/zcad061 (PMC10782916; doi:10.1093/narcan/zcad061)
Supplement: zcad061_Supplemental_Files [file zcad061_supplemental_files.zip › Figure_S5.pdf]

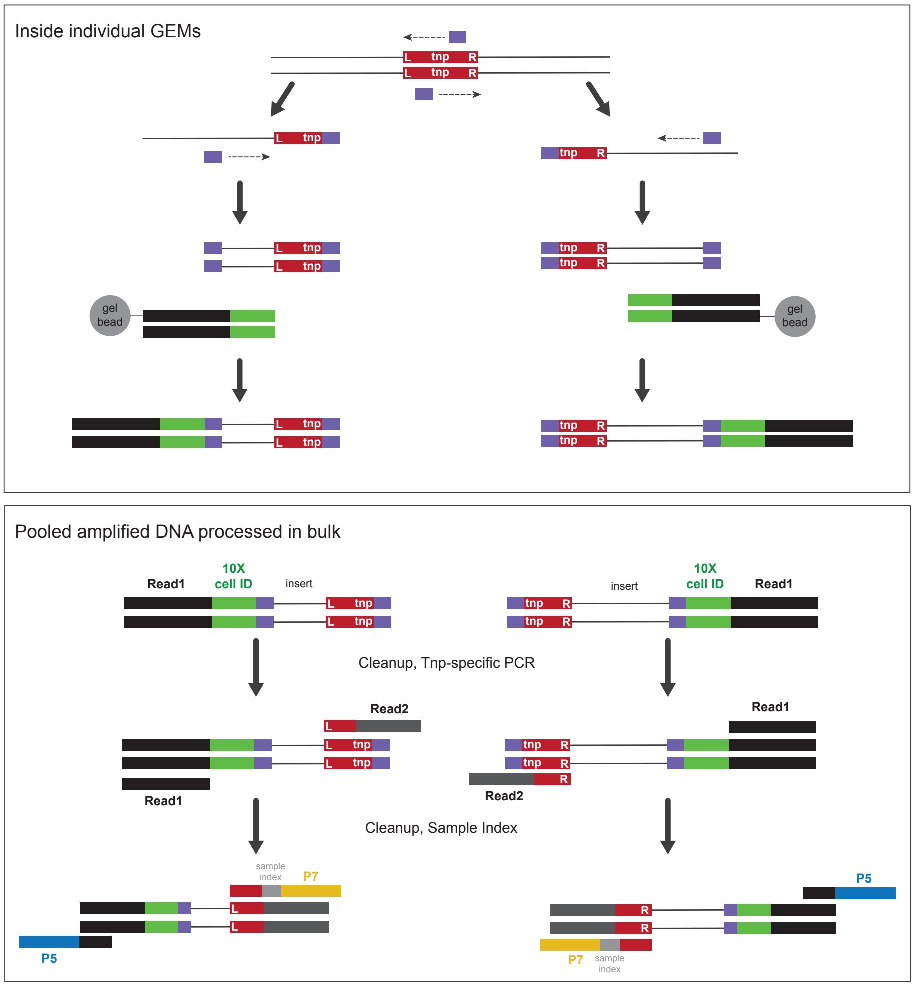

Supplemental Figure 5. Overview of modified method utilizing 10X Genomics single cell CNV approach to profile transposon insertion sites. The standard protocol was modified to specifically amplify transposon-genomic DNA junction fragments from individual cells. The portion of the method performed inside individual GEMs (Gel Bead-in-Emulsion) was carried out as described in the manufacturer's protocol (top panel). The protocol was customized after the GEMs were disrupted and cell-barcoded DNA fragments were isolated (bottom panel). Instead of adding the Read2 primer sequence by ligation, we performed a PCR reaction using a transposon-specific primer tagged with the Read2 sequence along with the standard Read1 primer. Finally, the P5 and P7 Illumina sequence adaptors were added using the standard sample indexing step of the protocol (see 10X Genomics protocol CG000153.RevC for overview of standard workflow).
