# Supplementary material for: Single-cell genomics analysis reveals complex genetic interactions in an in vivo model of acquired BRAF inhibitor resistance
Source: NAR Cancer. 2024 Jan 11;6(1):zcad061. doi: 10.1093/narcan/zcad061 (PMC10782916; doi:10.1093/narcan/zcad061)
Supplement: zcad061_Supplemental_Files [file zcad061_supplemental_files.zip › Figure_S6.pdf]

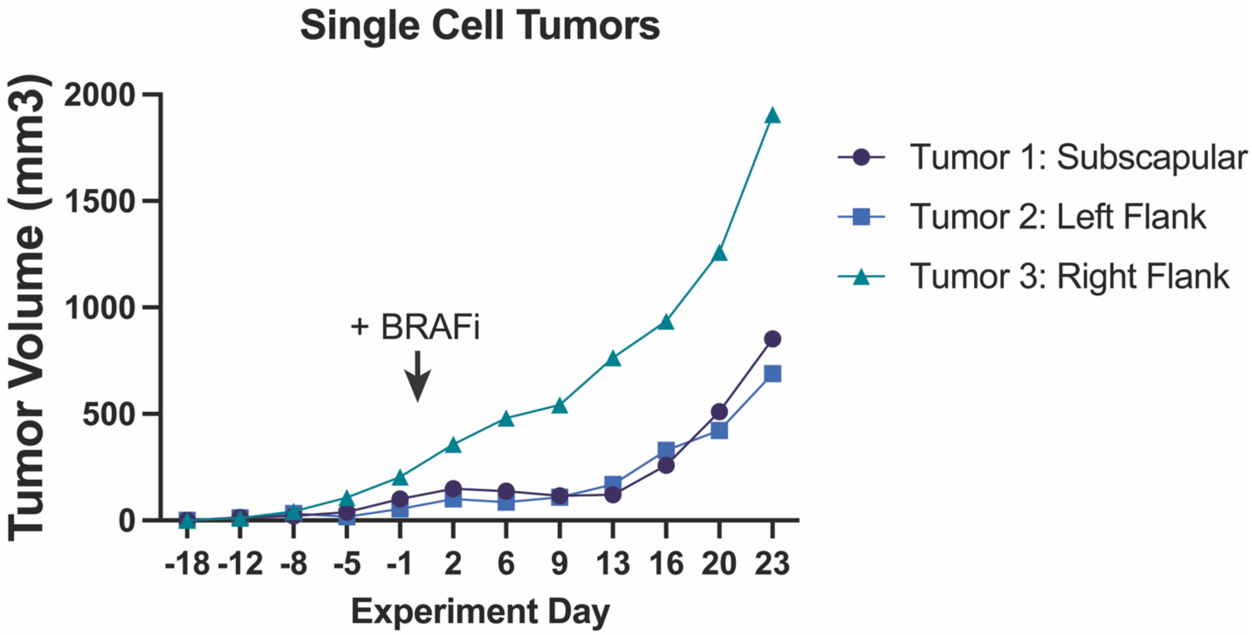

Supplemental Figure 6. Single-cell tumor growth characteristics. Three tumors were selected for single-cell analysis. The tumors were collected from the Subscapular, Left Flank, or Right Flank locations from independent mice.
