# Supplementary material for: Single-cell genomics analysis reveals complex genetic interactions in an in vivo model of acquired BRAF inhibitor resistance
Source: NAR Cancer. 2024 Jan 11;6(1):zcad061. doi: 10.1093/narcan/zcad061 (PMC10782916; doi:10.1093/narcan/zcad061)
Supplement: zcad061_Supplemental_Files [file zcad061_supplemental_files.zip › Figure_S7.pdf]

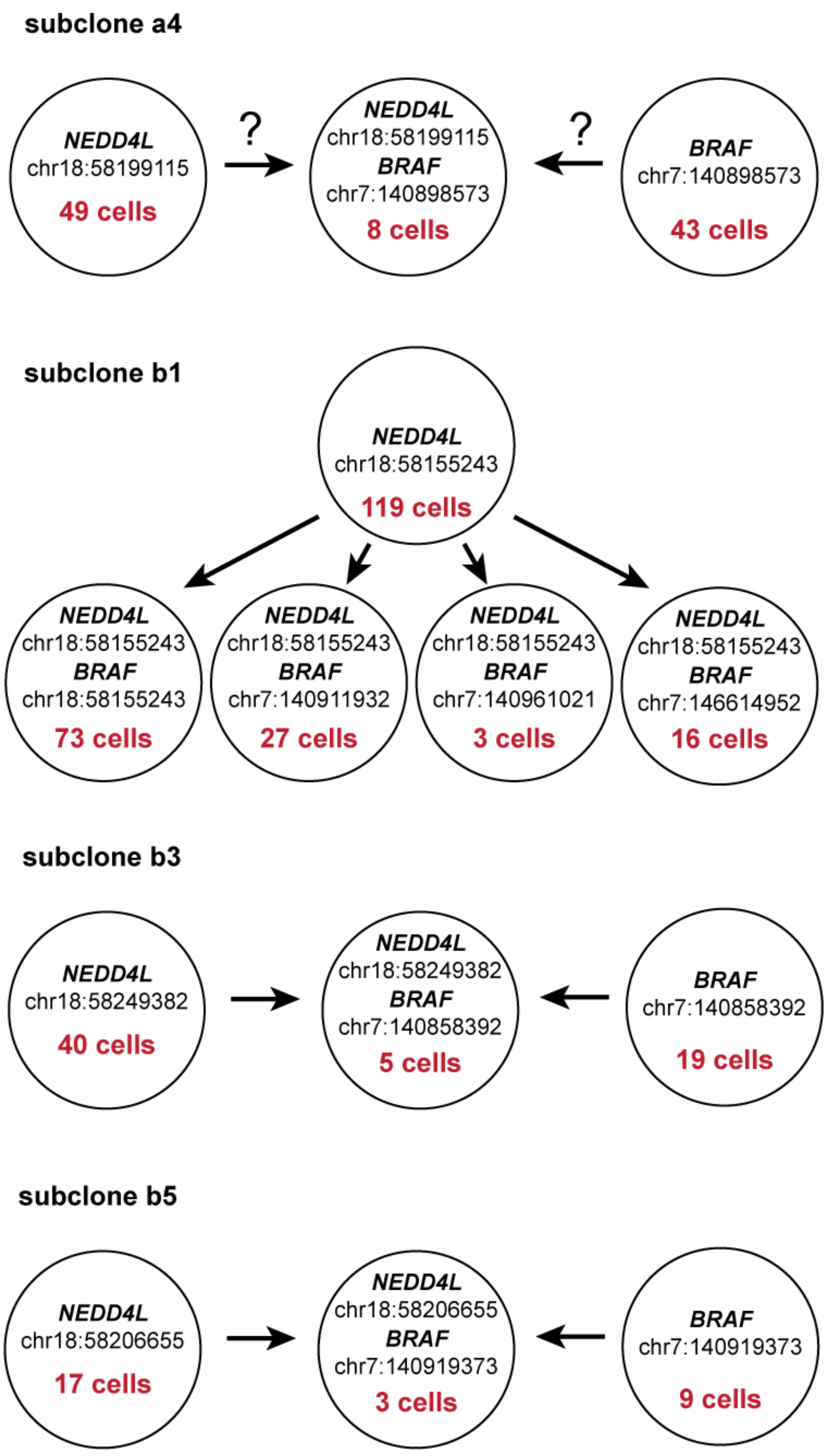

Supplemental Figure 7. Single cell analysis of individual subclones reveals details of *BRAF* and *NEDD4L* insertion events. Incomplete coverage leads to false negative detection of specific transposon insertion events. However, the order of mutation acquisition can be inferred by evaluating the number of cells with insertions in each gene, in most cases.
