# Supplementary material for: Single-cell genomics analysis reveals complex genetic interactions in an in vivo model of acquired BRAF inhibitor resistance
Source: NAR Cancer. 2024 Jan 11;6(1):zcad061. doi: 10.1093/narcan/zcad061 (PMC10782916; doi:10.1093/narcan/zcad061)
Supplement: zcad061_Supplemental_Files [file zcad061_supplemental_files.zip › Figure_S8.pdf]

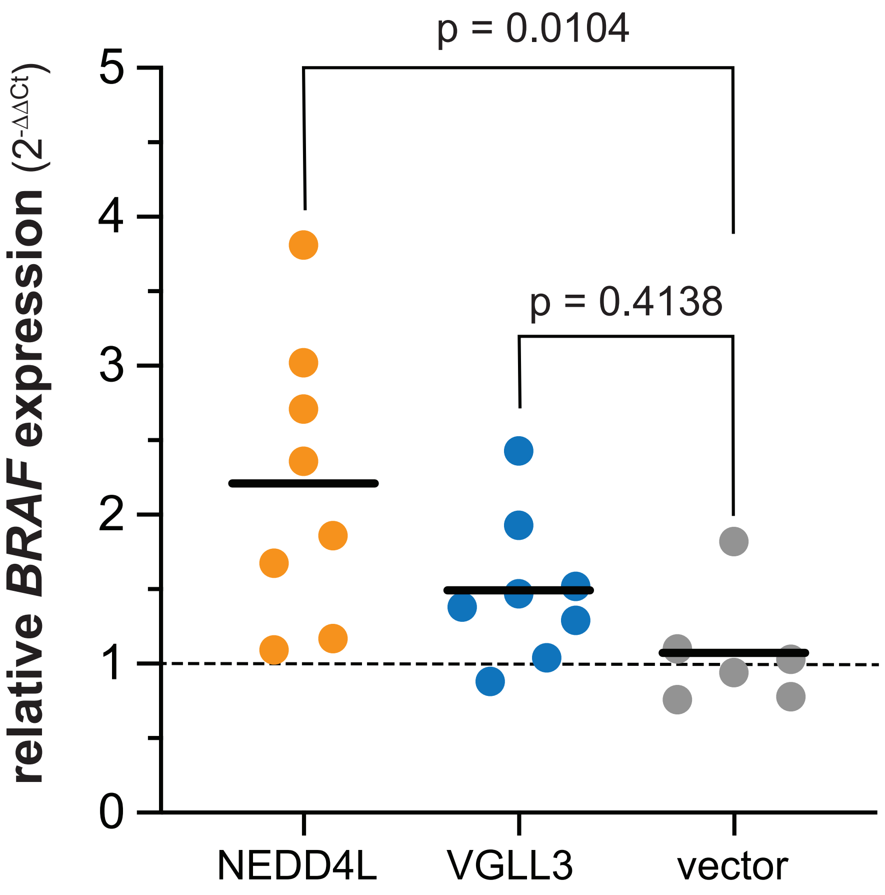

Supplemental Figure 8. Quantitative RT-PCR analysis of *BRAF* expression in NEDD4L, VGLL3, or control A375 xenograft tumors. The results of the single cell analysis strongly suggested that over-expression of BRAF and NEDD4L drives increased BRAFi resistance. We speculated that spontaneous over-expression of endogenous *BRAF*, perhaps through gene amplification, could cooperate with transgenic NEDD4L to drive progression of tumors described in Figure 3. We performed RT-qPCR to determine the *BRAF* expression level in each tumor. The *BRAF* expression value was normalized to *TBP* expression for each sample. The normalized BRAF expression was then expressed relative to parental A375 cells (indicated by dotted line). The p-values indicate the result of an ANOVA test.
