# Supplementary material for: Single-cell genomics analysis reveals complex genetic interactions in an in vivo model of acquired BRAF inhibitor resistance
Source: NAR Cancer. 2024 Jan 11;6(1):zcad061. doi: 10.1093/narcan/zcad061 (PMC10782916; doi:10.1093/narcan/zcad061)
Supplement: zcad061_Supplemental_Files [file zcad061_supplemental_files.zip › Figure_S9.pdf]

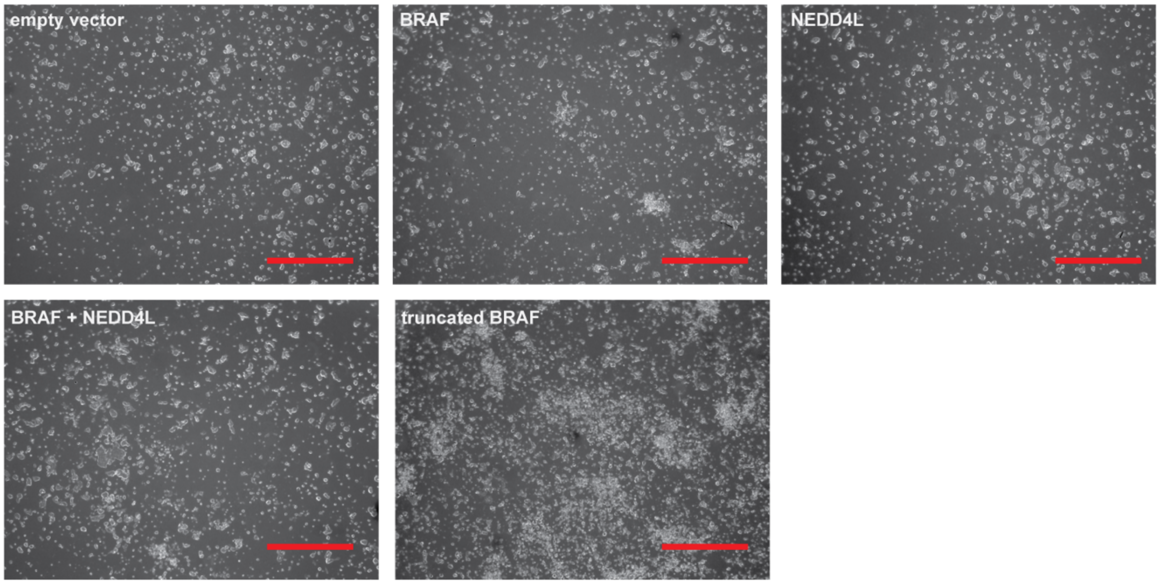

Supplementary Figure 9. Representative images of A375-derived populations growing on a collagen matrix in the presence of 0.5 μM PLX4720. [red scale bar = 1 milimeter]
