# Supplementary material for: Single-cell genomics analysis reveals complex genetic interactions in an in vivo model of acquired BRAF inhibitor resistance
Source: NAR Cancer. 2024 Jan 11;6(1):zcad061. doi: 10.1093/narcan/zcad061 (PMC10782916; doi:10.1093/narcan/zcad061)
Supplement: zcad061_Supplemental_Files [file zcad061_supplemental_files.zip › Table_S1.pdf]

Table S1. Results of gCIS analysis in BRAFi-treated A375 xenografts.

gene_symbol	chr	start (bp)	stop (bp)	strand	# of hits	# of TAs	# of samples	p-value	FDR	# in gene body [+,-]	# in promoter [+,-]	skewness	kurtosis	functional prediction
BRAF	chr7	140,719,330	140,924,711	-	438	386	77	1.00E-300	1.78E-298	379 [6,373]	59 [2,57]	0.13651	-0.78390	over-expression
NEDD4L	chr18	58,044,361	58,401,539	+	189	179	72	1.00E-300	1.78E-298	182 [177,5]	7 [5,2]	0.27787	-0.70544	over-expression
ABCG2	chr4	88,090,268	88,231,417	-	75	74	43	1.00E-300	1.78E-298	75 [1,74]	0 [0,0]	0.28473	-0.07543	over-expression
VGLL3	chr3	86,937,972	86,991,293	-	68	61	44	1.00E-300	1.78E-298	17 [2,15]	51 [1,50]	0.33291	1.53167	over-expression
CDC27P11	chr21	8,466,839	8,470,663	-	51	38	34	1.00E-300	1.78E-298	50 [29,21]	1 [1,0]	-0.78483	-0.01991	disruption
MAP3K1	chr5	56,815,072	56,896,152	+	19	18	18	7.32E-39	8.82E-37	16 [16,0]	3 [3,0]	0.62898	-0.52925	over-expression
ITCH	chr20	34,363,234	34,511,773	+	20	17	16	5.89E-43	7.33E-41	14 [13,1]	6 [5,1]	-1.54059	3.32316	over-expression
BCL6	chr3	187,721,376	187,745,725	-	19	17	15	2.17E-59	3.00E-57	5 [0,5]	14 [0,14]	0.79499	-0.59590	over-expression
VAV1	chr19	6,772,667	6,857,366	+	16	15	16	1.55E-50	2.06E-48	14 [12,2]	2 [2,0]	1.27467	1.07829	over-expression
FGD4	chr12	32,399,527	32,646,050	+	15	12	15	1.22E-14	1.23E-12	13 [12,1]	2 [1,1]	0.06347	-1.28802	over-expression
MAP3K13	chr3	185,282,937	185,489,094	+	11	10	11	3.96E-08	3.90E-06	11 [11,0]	0 [0,0]	1.01121	0.40893	over-expression
MCF2L	chr13	112,894,377	113,098,550	+	9	9	9	8.74E-15	9.06E-13	9 [8,1]	0 [0,0]	1.02700	-0.72979	over-expression
RAF1	chr3	12,583,600	12,664,201	-	10	7	10	1.79E-15	1.91E-13	10 [0,10]	0 [0,0]	2.76790	5.84923	over-expression
