# Supplementary material for: Single-cell genomics analysis reveals complex genetic interactions in an in vivo model of acquired BRAF inhibitor resistance
Source: NAR Cancer. 2024 Jan 11;6(1):zcad061. doi: 10.1093/narcan/zcad061 (PMC10782916; doi:10.1093/narcan/zcad061)
Supplement: zcad061_Supplemental_Files [file zcad061_supplemental_files.zip › Table_S2.pdf]

Table S2. Results of gCIS analysis of untreated A375 xenografts.

gene_symbol	chr	start (bp)	stop (bp)	strand	# of hits	# of TAs	# of samples	p-value	FDR	# in gene body [+,-]	# in promoter [+,-]	skewness	kurtosis	functional prediction
NEDD4L	chr18	58,044,361	58,401,539	+	61	60	10	1.00E-300	6.11E-299	57 [55,2]	4 [3,1]	0.5021	-0.9726	over-expression
VGLL3	chr3	86,937,972	86,991,293	-	17	17	8	4.50E-116	2.58E-114	7 [0,7]	10 [0,10]	0.5020	1.2861	over-expression
CDC27P11	chr21	8,466,839	8,470,663	-	12	11	7	1.00E-300	6.11E-299	11 [5,6]	1 [0,1]	-0.7834	-0.3686	disruption
HMGA2	chr12	65,824,459	65,966,291	+	11	5	3	2.95E-41	1.13E-39	11 [11,0]	0 [0,0]	3.0148	7.0897	over-expression
MECOM	chr3	169,083,498	169,663,775	-	8	8	6	8.31E-08	6.81E-07	8 [1,7]	0 [0,0]	-0.9089	0.0077	over-expression
TSPAN11	chr12	30,926,439	31,016,502	+	7	4	3	2.94E-26	8.18E-25	0 [0,0]	7 [7,0]	2.2621	3.1286	over-expression
ITCH	chr20	34,363,234	34,511,773	+	7	7	6	3.09E-19	7.66E-18	6 [6,0]	1 [1,0]	1.1985	0.4153	over-expression
TEAD1	chr11	12,674,421	12,944,737	+	6	6	6	2.36E-09	2.43E-08	4 [3,1]	2 [2,0]	-1.4261	0.8386	over-expression
SMAP1	chr6	70,667,770	70,862,015	+	6	3	5	4.26E-10	4.94E-09	2 [2,0]	4 [4,0]	-0.4355	-1.0073	over-expression
MYOF	chr10	93,306,428	93,482,496	-	5	4	2	6.48E-09	6.13E-08	1 [0,1]	4 [0,4]	1.1395	-0.3223	over-expression
SPTLC3	chr20	13,008,978	13,166,764	+	5	3	3	5.70E-08	4.84E-07	5 [5,0]	0 [0,0]	-1.1122	0.4258	over-expression
