# Supplementary material for: Single-cell genomics analysis reveals complex genetic interactions in an in vivo model of acquired BRAF inhibitor resistance
Source: NAR Cancer. 2024 Jan 11;6(1):zcad061. doi: 10.1093/narcan/zcad061 (PMC10782916; doi:10.1093/narcan/zcad061)
Supplement: zcad061_Supplemental_Files [file zcad061_supplemental_files.zip › Table_S3.pdf]

Table S3. Adjacency matrix for sample 1, corresponds to Figure 4a.

clone	site	chr13:97310848	chr14:23606188	chr14:31125889	chr8:52295207	chr11:127095041	chr8:58928525	chr19:35969773	chr10:62568084	chr7:140894519	chr7:142316524	chr7:142560054	chr8:125213754	chr7:140899103	chr7:140807432	chr8:94385520	chr15:85577945	chr7:140795214	chr7:139846468	chr2:113470934	chr16:55726140
a1	chr13:97310848		[illegible]	91	84	77	[illegible]	[illegible]	86	89	44	25	5	13		2					
a1	chr14:23606188	[illegible]		75	88	84	[illegible]	83	[illegible]	85	55	15	5	11	2						
a1	chr14:31125889	91	75		82	68	78	75	85	74	39	20	4	10	2						
a1	chr8:52295207	84	88	82		87	80	76	82	75	53	17	5	9							
a1	chr11:127095041	77	84	68	87		78	84	70	62	51	17	4	8	2						
a1	chr8:58928525	[illegible]	94	78	80	78		69	73	82	53	21	4	13	4						
a1	chr19:35969773	[illegible]	83	75	76	84	69		74	69	46	16	4	12							
a1	chr10:62568084	86	[illegible]	85	82	70	73	74		88	53	22	6	9							
a1	chr7:140894519	89	85	74	75	62	82	69	88		61	21	2								
a1	chr7:142316524	44	55	39	53	51	53	46	53	61		2			2						
a1	chr7:142560054	25	15	20	17	17	21	16	22	21	2										
a1	chr8:125213754	5	5	4	5	4	4	4	6	2											
a1	chr7:140899103	13	11	10	9	8	13	12	9												
a1	chr7:140807432		2	2		2	4				2										
a1	chr8:94385520	2																			
a2	chr15:85577945																	21	51	12	
a2	chr7:140795214																21		26	6	
a2	chr7:139846468																51	26		7	3
a2	chr2:113470934																12	6	7		
a2	chr16:55726140																		3		

[illegible]
