# Supplementary material for: Single-cell genomics analysis reveals complex genetic interactions in an in vivo model of acquired BRAF inhibitor resistance
Source: NAR Cancer. 2024 Jan 11;6(1):zcad061. doi: 10.1093/narcan/zcad061 (PMC10782916; doi:10.1093/narcan/zcad061)
Supplement: zcad061_Supplemental_Files [file zcad061_supplemental_files.zip › Table_S5.pdf]

Table S5. Adjacency matrix for sample 3, corresponds to Figure 4c.

clone	site	chr6:149788923	chr15:70847289	chr1:7709137	chr3:162708695	chr5:60749592	chr5:63009794	chr9:23185097	chr1:100785525	chr12:81944310	chr7:84637813	chr1:200217837	chr18:2488202	chr5:57616046	chr20:14941543	chr3:138996919	chr3:86981877	chr22:34473404	chr7:140912101	chr11:61455973	chr6:31330601	chr16:59751549	chr5:39148170	chr16:7567234	chr5:61109893	chr17:33567914
c1	chr6:149788923		13	20	13	22	17	12	19	15	16	19	[illegible]	15	15	16	14	13	4	14	16	10	5	7	6	
c1	chr15:70847289	13		18	10	14	14	12	8	9	11	11	16	12	10	8	11	8	2	9	8	8	6	6	2	
c1	chr1:7709137	20	18		18	22	16	13	18	13	15	11	17	14	13	16	20	16	4	12	13	8	9	3	6	
c1	chr3:162708695	13	10	18		12	10	8	10	10	9	8	13	8	8	7	11	11	4	9	11	3	4	5	2	
c1	chr5:60749592	22	14	22	12		19	14	17	13	18	13	14	14	11	16	14	7	3	7	18	8	6	7	2	
c1	chr5:63009794	17	14	16	10	19		17	19	19	16	11	20	17	15	13	13	17	5	11	13	11	7	13	3	
c1	chr9:23185097	12	12	13	8	14	17		14	9	12	11	12	14	10	12	10	10	5	8	14	9	7	10		2
c1	chr1:100785525	19	8	18	10	17	19	14		9	17	12	16	11	10	12	13	14	5	6	12	8	7	12	7	
c1	chr12:81944310	15	9	13	10	13	19	9	9		12	9	15	12	6	8	14	11	3	9	9	7	3	11		
c1	chr7:84637813	16	11	15	9	18	16	12	17	12		17	21	18	16	16	20	12	4	14	9	7	5	14	6	
c1	chr1:200217837	19	11	11	8	13	11	11	12	9	17		15	14	11	14	19	9	5	11	8	5	3	10	5	
c1	chr18:2488202	[illegible]	16	17	13	14	20	12	16	15	21	15		19	12	12	17	13	4	15	9	5	3	10	9	
c1	chr5:57616046	15	12	14	8	14	17	14	11	12	18	14	19		6	10	12	13	3	9	7	7	5	7	5	2
c1	chr20:14941543	15	10	13	8	11	15	10	10	6	16	11	12	6		12	13	7	2	5	4	6	4	9	3	
c1	chr3:138996919	16	8	16	7	16	13	12	12	8	16	14	12	10	12		12	13	4	15	11	5	5	9	10	
c1	chr3:86981877	14	11	20	11	14	13	10	13	14	20	19	17	12	13	12		14	3	7	3	5	4	6	3	
c1	chr22:34473404	13	8	16	11	7	17	10	14	11	12	9	13	13	7	13	14			13	7	9	5	6	6	
c1	chr7:140912101	4	2	4	4	3	5	5	5	3	4	5	4	3	2	4	3			4	2					
c1	chr11:61455973	14	9	12	9	7	11	8	6	9	14	11	15	9	5	15	7	13	4		9	6	2	2	4	
c1	chr6:31330601	16	8	13	11	18	13	14	12	9	9	8	9	7	4	11	3	7	2	9		3	6	10	3	
c1	chr16:59751549	10	8	8	3	8	11	9	8	7	7	5	5	7	6	5	5	9		6	3		4	7		
c1	chr5:39148170	5	6	9	4	6	7	7	7	3	5	3	3	5	4	5	4	5		2	6	4		3	2	
c1	chr16:7567234	7	6	3	5	7	13	10	12	11	14	10	10	7	9	9	6	6		2	10	7	3		2	
c1	chr5:61109893	6	2	6	2	2	3		7		6	5	9	5	3	10	3	6		4	3		2	2		
c2	chr17:33567914							2						2												

clone	site	chr1:1857020	chr10:57693266	chr7:81832726	chr1:185906634	chr10:99276473
c2	chr1:1857020		18	12	16	14
c2	chr10:57693266	18		13	15	15
c2	chr7:81832726	12	13		11	8
c2	chr1:185906634	16	15	11		16
c2	chr10:99276473	14	15	8	16	

clone	site	chr20:37824842	chr1:182428705	chr15:76079816	chr16:83106886	chr8:116697871	chr6:80730376	chr13:70644960	chrX:27372782	chrX:74274872	chr12:33763008	chr8:76502017	chr9:35875905	chr1:220727598	chrX:97219849	chr19:47816644	chr10:114372678	chr1:87669159	chr19:5624287	chr6:134944953	chr17:78847868	chr4:21977720	chr2:217588100	chr7:26848819	chr2:220877128	chr3:12605158	chr11:122496218	chr8:88346960	chr3:35986570	chr20:5558094	chr1:111960773	chr1:20601991	chr7:149158904	chr5:50515217	chr1:216173795	chr7:152378675	chr5:34212057
c3	chr20:37824842		7	9	9	7	11	11	7	8	6	10	7	3	5	5	7	5	6	2	7	3	7	5	6	2	3	4	4	4	3	4	3	2	3	5	6
c3	chr1:182428705	7		4	4	5	4	5	5	3	3	5	4	2	4		5	4	4	3	3	2	5	2	3		2			4	4	2	4				2
c3	chr15:76079816	9	4		4	5	3	2	2	3	2	4	3		2		4	4	3	2	3		3	2	2				2	5	4		2	2	2		3
c3	chr16:83106886	9	4	4		5	2	5	7	3	3	5	7		3	5	7	2	2	2	5	4	3	2	3				3	2	2	3				2	
c3	chr8:116697871	7	5	5	5		8	8	14	10	9	9	7	8	3	3	4	4	5	5	5	3	3	2	3		3	5	4	6	5	2	2		2	2	2
c3	chr6:80730376	11	4	3	2	8		6	12	11	9	9	7	6	7	4	5	3	3	3	5	4	7	4	4	4	2	3	5	4	4	6		2	4	2	4
c3	chr13:70644960	11	5	2	5	8	6		9	8	6	6	8	8	5	7	6	4	4	3	2	4	4	3	4	3	4	3	4		4		2	3	4	3	2
c3	chrX:27372782	7	5	2	7	14	12	9		11	6	7	5	8	4	5	7	6	5	4	7	5	7	4	5	4	6	2		5	6	5		2	2	4	3
c3	chrX:74274872	8	3	3	3	10	11	8	11		5	6	6	8	6	4	4	6	7	6	6	5	8	5	6	4	5	4	4	3	2	3	3	2	2	2	5
c3	chr12:33763008	6	3	2	3	9	9	6	6	5		8	10	5	4	4	7	4	3	4	2	2	6	3	2	3	5	5		6	5	5	2		2	3	2
c3	chr8:76502017	10	5	4	5	9	9	6	7	6	8		7	7	7	6	5	3	2	3	5	2	5	6	3	5	4	6	3	4	4	4	4	3		4	2
c3	chr9:35875905	7	4	3	7	7	7	8	5	6	10	7		5	3	7	6	3	3	5	3	2	4	2	3	4		5	3	3	3	6	2	2	3		
c3	chr1:220727598	3	2			8	6	8	8	8	5	7	5		6	5	4		5	3	5		3	2	2	4	3		5	4	3	4	2	2	2		2
c3	chrX:97219849	5	4	2	3	3	7	5	4	6	4	7	3	6		5	4	3	5	2		4	4	4	2	4	3		5	2	2	3		3		2	
c3	chr19:47816644	5			5	3	4	7	5	4	4	6	7	5	5		3	4	4	2	5	4	3	3		2	2	3	3	5	5	4	2		3	4	
c3	chr10:114372678	7	5	4	7	4	5	6	7	4	7	5	6	4	4	3		6	6	4	5	3	3	2	2	2	4	3	2	4	7	5		2	2	2	
c3	chr1:87669159	5	4	4	2	4	3	4	6	6	4	3	3		3	4	6		6	4	6	4		3	3	2		2		5	5	3	3		2	3	
c3	chr19:5624287	6	4	3	2	5	3	4	5	7	3	2	3	5	5	4	6	6		5	5	2	5	4	3		2		4	3	3		4				2
c3	chr6:134944953	2	3	2	2	5	3	3	4	6	4	3	5	3	2	2	4	4	5		3	2	3	2	4	2	2	2	2	3	2	4	3		2		
c3	chr17:78847868	7	3	3	5	5	5	2	7	6	2	5	3	5		5	5	6	5	3		4	3	2	3	2	2	2	2	6	2	5	2			8	
c3	chr4:21977720	3	2		4	3	4	4	5	5	2	2	2		4	4	3	4	2	2	4		2	3	2		2				2		2			4	
c3	chr2:217588100	7	5	3	3	3	7	4	7	8	6	5	4	3	4	3	3		5	3	3	2		4	3	3	4	2	2			8	6	5		2	6
c3	chr7:26848819	5	2	2	2	2	4	3	4	5	3	6	2	2	4	3	2	3	4	2	2	3	4		4	4	3	4				3	3	2		3	2
c3	chr2:220877128	6	3	2	3	3	4	4	5	6	2	3	3	2	2		2	3	3	4	3	2	3	4		3	2	2	2			2	2			2	2
c3	chr3:12605158	2					4	3	4	4	3	5	4	4	4	2	2	2		2	2		3	4	3		2			2		3	2	2		4	2
c3	chr11:122496218	3	2			3	2	4	6	5	5	4		3	3	2	4		2	2	2	2	4	3	2	2		3	2	4		5	3	3			2
c3	chr8:88346960	4				5	3	3	2	4	5	6	5			3	3	2		2	2		2	4	2		3			3	5	4	3	2		2	
c3	chr3:35986570	4		2	3	4	5	4		4		3	3	5	5	3	2		4	2	2		2		2		2			6	2	2					
c3	chr20:5558094	4	4	5	2	6	4		5	3	6	4	3	4	2	5	4	5	3	3	6					2	4	3	6		6	4	3		2	2	
c3	chr1:111960773	3	4	4	2	5	4	4	6	2	5	4	3	3	2	5	7	5	3	2	2	2						5	2	6		4			2		
c3	chr1:20601991	4	2		3	2	6		5	3	5	4	6	4	3	4	5	3		4	5		8	3	2	3	5	4	2	4	4			2	3	2	2
c3	chr7:149158904	3	4	2		2		2		3	2	4	2	2		2		3	4	3	2	2	6	3	2	2	3	3		3				2	2		2
c3	chr5:50515217	2		2			2	3	2	2		3	2	2	3		2						5	2		2	3	2				2	2				2
c3	chr1:216173795	3		2		2	4	4	2	2	2		3	2		3	2	2		2										2	2	3	2				2
c3	chr7:152378675	5			2	2	2	3	4	2	3	4			2	4	2	3			8	4	2	3	2	4		2		2		2					
c3	chr5:34212057	6	2	3		2	4	2	3	5	2	2		2					2				6	2	2	2	2					2	2	2	2		

clone	site	chr13:61929426	chr3:24149562	chr18:58186541	chr5:33955459	chr3:117681972	chr18:72052337	chr3:136635917	chr17:65358301	chr20:58394255
c4	chr13:61929426		4	6	12	12	8	9	11	6
c4	chr3:24149562	4		4	8	6	4	5	4	3
c4	chr18:58186541	6	4		8	5	7	8	5	3
c4	chr5:33955459	12	8	8		8	6	7	8	4
c4	chr3:117681972	12	6	5	8		5	5	7	5
c4	chr18:72052337	8	4	7	6	5		4	9	3
c4	chr3:136635917	9	5	8	7	5	4		2	
c4	chr17:65358301	11	4	5	8	7	9	2		2
c4	chr20:58394255	6	3	3	4	5	3		2	

clone	site	chr8:52697864	chr10:9128518	chr19:19399629	chr11:118442856	chr2:88304373	chr7:107582323	chr22:40171888	chr13:88225910
c5	chr8:52697864		6	3	4	7	3	2	4
c5	chr10:9128518	6		5	2	7	2	3	5
c5	chr19:19399629	3	5			5	2	3	
c5	chr11:118442856	4	2			5	3	3	4
c5	chr2:88304373	7	7	5	5		4	2	
c5	chr7:107582323	3	2	2	3	4			
c5	chr22:40171888	2	3	3	3	2			
c5	chr13:88225910	4	5		4				

clone	site	chr5:141813672	chrX:10055587
c6	chr5:141813672		12
c6	chrX:10055587	12	
