# Supplementary material for: Single-cell genomics analysis reveals complex genetic interactions in an in vivo model of acquired BRAF inhibitor resistance
Source: NAR Cancer. 2024 Jan 11;6(1):zcad061. doi: 10.1093/narcan/zcad061 (PMC10782916; doi:10.1093/narcan/zcad061)
Supplement: zcad061_Supplemental_Files [file zcad061_supplemental_files.zip › Table_S6.pdf]

Table S6. Primer sequences

**Primer name**	**sequence**
NEDD4L fwd	5′-ATGGAGCGACCCTATACATTTAAGGACTTT-3’
NEDD4L rev	5′-TTAATCCACCCC TTCAAATCCTTGAGCATT-3’
VGLL3 fwd	5′-ATGAGTTGTGCGGAGGTGATGTAT-3’
VGLL3 rev	5′-TCAAGATCTCGCCATTGCACTCC-3’
BRAF RT exon 12 fwd	5’-ATGGTGATGTGGCAGTGAAA-3’
BRAF RT exon 13 rev	5’-ATGAAGAGTAGGATATTCACATGTCG-3’
TBP RT fwd	5’-GTGGGGAGCTGTGATGTGAA-3’
TBP RT rev	5’-TGCTCTGACTTTAGCACCTGT-3’
10X transposon primer	5’GTGACTGGAGTTCAGACGTGTGCTCTTCCGATCTTGTATGTAAACTTCCGACTTCAACTG-3’
